# The role of breast-feeding in infant immune system: a systems perspective on the intestinal microbiome

**DOI:** 10.1186/s40168-015-0104-7

**Published:** 2015-09-24

**Authors:** Paurush Praveen, Ferenc Jordan, Corrado Priami, Melissa J. Morine

**Affiliations:** The Microsoft Research-University of Trento Centre for Computational and Systems Biology, 38068 Rovereto, Italy; Department of Mathematics, University of Trento, 38100 Povo, Italy

## Abstract

**Background:**

The human intestinal microbiota changes from being sparsely populated and variable to possessing a mature, adult-like stable microbiome during the first 2 years of life. This assembly process of the microbiota can lead to either negative or positive effects on health, depending on the colonization sequence and diet. An integrative study on the diet, the microbiota, and genomic activity at the transcriptomic level may give an insight into the role of diet in shaping the human/microbiome relationship. This study aims at better understanding the effects of microbial community and feeding mode (breast-fed and formula-fed) on the immune system, by comparing intestinal metagenomic and transcriptomic data from breast-fed and formula-fed babies.

**Results:**

We re-analyzed a published metagenomics and host gene expression dataset from a systems biology perspective. Our results show that breast-fed samples co-express genes associated with immunological, metabolic, and biosynthetic activities. The diversity of the microbiota is higher in formula-fed than breast-fed infants, potentially reflecting the weaker dependence of infants on maternal microbiome. We mapped the microbial composition and the expression patterns for host systems and studied their relationship from a systems biology perspective, focusing on the differences.

**Conclusions:**

Our findings revealed that there is co-expression of more genes in breast-fed samples but lower microbial diversity compared to formula-fed. Applying network-based systems biology approach via enrichment of microbial species with host genes revealed the novel key relationships of the microbiota with immune and metabolic activity. This was supported statistically by data and literature.

**Electronic supplementary material:**

The online version of this article (doi:10.1186/s40168-015-0104-7) contains supplementary material, which is available to authorized users.

## Background

The intestinal microbiota and its human host develop a strong mutual relationship [1]. Several vital functions (e.g., metabolism and innate and adaptive immunity) are affected by the intestinal microbiota. The microbiota of older individuals displays greater inter-individual variation than that of younger adults [2, 3]. The infant intestinal microbiota is more variable in its composition and is less stable over time. In the first 2 years of life, the infant intestinal tract progresses from sterility (pre-natal colonization might influence the sterility [4]) to extremely dense colonization, ending with a mixture of microbes that is broadly very similar to that found in the adult intestine [4–6]. The Human Microbiome Project [7] has investigated the stability and individual-level variability of human microbiota depending on geography, environment, lifestyle, and other factors [4]. However, the assembly of the microbial community during infancy remains poorly understood despite being essential to human health [8, 9]. Despite the fact that bacterial cells outnumber the total number of human cells in the body, the human gut contains a surprisingly limited number of dominant enterotypes [10]. Starting primarily with facultative anaerobes, e.g., *Escherichia coli*, the microbial community is later diversified with anaerobes, e.g., *Bifidobacterium* and *Clostridium* [11]. The factors that affect the colonization process after birth include feeding, proboitic treatment, and environmental factors. An atypical intestinal colonization during the first weeks of life may alter the nutritional and immunological functions of the host microbiota [12] and increase susceptibility to immunological and metabolic diseases [13].

Despite the fact that bacterial cells outnumber the total number of human cells in the body, the human gut contains a surprisingly limited number of dominant enterotypes [10]. Starting primarily with facultative anaerobes, e.g., *E. coli*, the microbial community is later diversified with anaerobes, e.g., *Bifidobacterium* and *Clostridium* [11]. The factors that affect the colonization process after birth include feeding, proboitic treatment, and environmental factors. An atypical intestinal colonization during the first weeks of life may alter the nutritional and immunological functions of the host microbiota [12] and increase susceptibility to immunological and metabolic diseases [13].

A systems biology approach to study the relationships between the host and the microbiota requires the measurement of biomolecular activity within the host (e.g., via transcriptomic data) and the quantification of host microbiota under similar conditions [14]. The acquisition and diversity of the gut microbiota in term neonates have been the subject of several studies. Jiménez et al. [15] studied the microbiota at pre-birth stages in the umbilical cord, showing the possibility of a pre-natal colonization of infant gut. Then, Neu et al. [16] investigated the effect of mode of birth (caesarean and non-caesarean) showing the effects on the microbiota. The effect of diet on the gut microbiota in piglets and humans was demonstrated by Poroyko et al. [17] and Marques et al. [18], showing the differences between breast-fed and formula-fed piglets. Works by Eckburg et al. [19], Azad et al. [20, 21], and Thompson et al. [22] revealed the differences in the diversity of the microbiota in breast-fed and formula-fed infants. Schwartz et al. [14] made one of the first attempts to show the genome and transcriptome cross talk in breast-fed and formula-fed infants showing the effect of feeding mode on microbiome and transcriptome. Rogier et al. [23] demonstrated the role of breast-feeding on the microbiota and host gene expression at individual levels. However, a detailed cross-species study at the systems level is still missing. Molecular and metagenomic techniques together with rigorous computational analysis from systems biology perspective can approach this goal [24]. A systems perspective can add novel information on key relationships among the interacting individual components of this rich ecosystem, apart from their pure composition and abundance distribution [25]. Moreover, differences between the two feeding types can be quantified and visualized.

Our study aims to observe cross talk at microbial and transcript level, in order to understand the effect of feeding mode on the host system, on the microbiome, and on the interaction between the two. We present a detailed analysis of the data studied in [14], from systems biology perspective, to elucidate the relationship between the feeding mode, the microbiota, and host cell activities. The data come from samples under two feeding conditions: exclusively breast-feeding (BF) and exclusively formula-feeding (FF). The formula food was Enfamil^®^ LIPIL^®^: a commercially available formula.

Our analysis is an extension of the work of Schwartz et al. [14] with the systems biology point of view in terms of relation between the feeding mode, the microbiota, and host cell activities. We identify relations between the microbial species and host genes using the enrichment analysis of microbiome with bibliographic data, followed by the verification of the relations via bivariate analysis on experimental data. Finally, we present a system level overview of a host gene-microbiota relationship at network level in order to distinguish the infant systems based on the modes of feeding.

## Results

Our results indicated the effect of feeding mode both on the microbiota and the expression patterns of certain genes.

### The microbiota

**Fig. 1:**
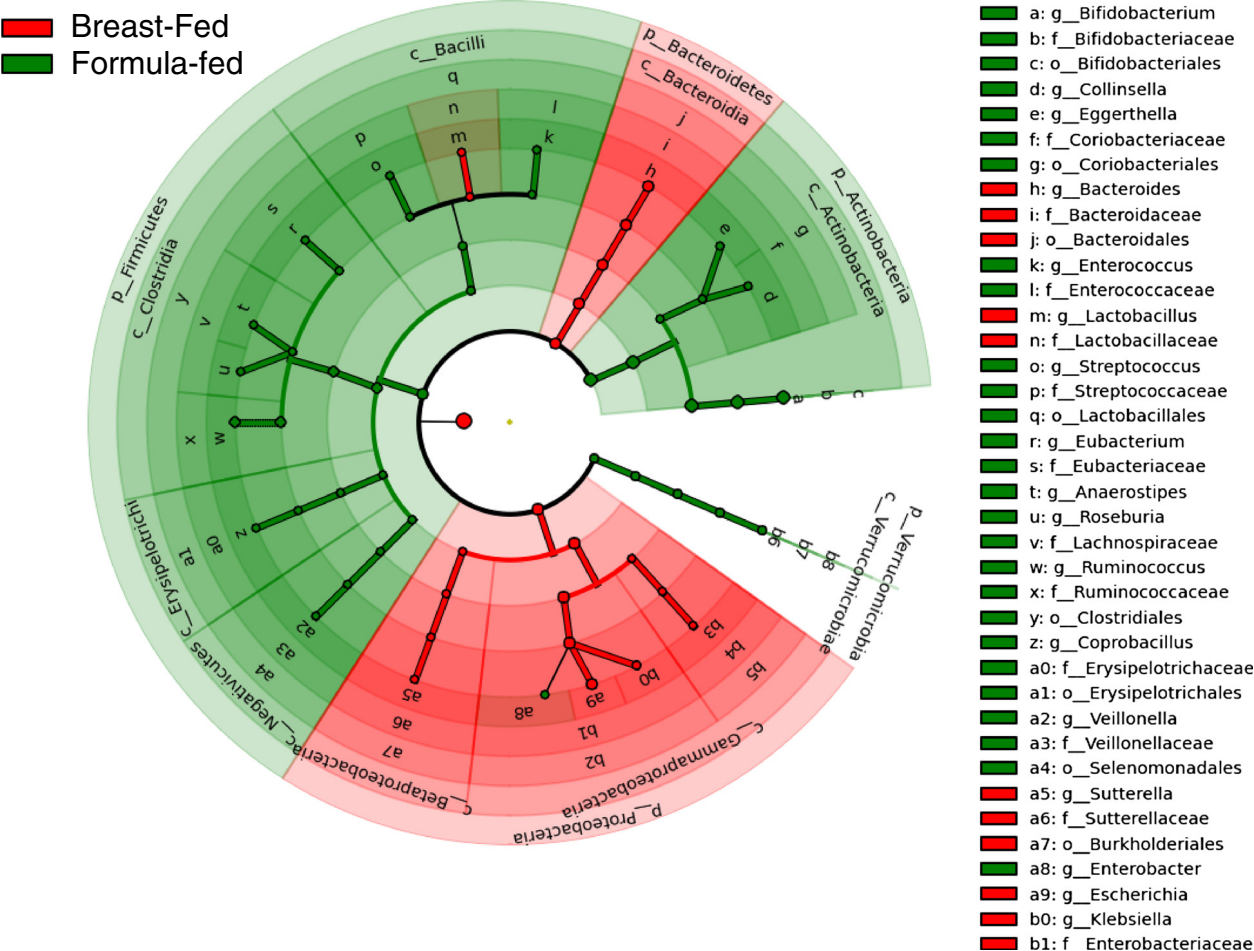
The LEfSe plot for clades of the microbiota under breast-fed (BF) and formula-fed (FF) conditions. The cladograms report the taxa (highlighted by *small circles* and by *shading*) showing different abundance values (according to LEfSe). Colors of circle and shading indicate the microbial lineages that are enriched within corresponding samples. LEfSe highlights several genus-level clades, e.g., the class *Bacilli* is under-abundant in BF samples with an otherwise over-abundant *Lactobacillus* lineage (indicated with a *red shade over green* for indices *m* and *n* (see adjacent legend)). A contrary example can be seen in case of *Enterobacter* (indexed as *a8*)

The microbiota under two feeding conditions showed enrichments with different bacterial lineages (Fig. 1). The microbiota in both feeding conditions had higher fraction of anaerobes compared to facultative anaerobes. The analysis of bacterial phyla in the sample shared high agreement with the findings of Schwartz et al. [14] with the *Actinobacteria* showing prominence under both feeding conditions (see Additional file 1). The *Firmicutes* associated with energy resorption and obesity [26] were more abundant in FF samples than in BF samples (see Additional file 1). We also computed the diversity of the microbiota (see Additional file 1) in terms of Shannon index [27].

**Fig. 2:**
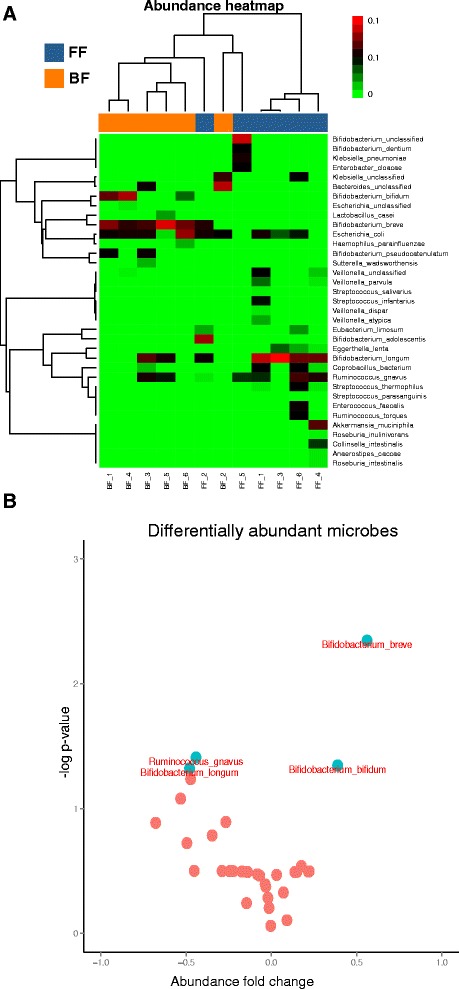
**a** The heatmap showing the abundance of microbes at species level, in breast-fed and formula-fed infants. *Green* and *red shades* indicate lower and higher percent abundances, respectively, with species along the Y-axis and samples along X-axis. The clustering was performed with the “Ward” method based on Pearson scores. **b** Scatter plot representing the log *p* values (Y-axis) and fold changes (X-axis) for microbial abundance to detect the differentially abundant bacterial species. The *blue-green circles* indicate the differentially abundant microbial species under FF and BF conditions

The microbial diversity (see Additional file 1) in our analysis partially differ from Schwartz et al. potentially due to the use of a more updated marker sequence-based database [28] for Bowtie 2 [29]. The filtering of the microbiota results to the species level showed 35 taxonomic species (with 4 of the data points showing equal relatedness with more that one species, hence designated as unclassified (see Additional file 1)). The metagenomic features (microbial abundances) provided a clear distinction between the two feeding types (Fig. 2a). We performed a differential abundance analysis (see “Methods”) that revealed four species to be differentially abundant in the samples given the two feeding types (Fig. 2b). The four species included three *Bifidobacterium* species together with *Ruminococcus gnavus*. The gap in diversity also widened for the species level enrichment (see Additional file 1).

**Fig. 3:**
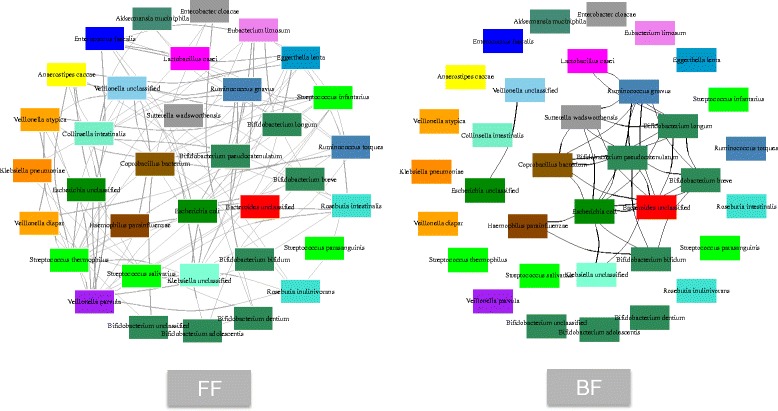
The microbiome co-abundance networks based on Bray-Curtis similarity under FF and BF conditions. Node color indicates the genera of the organisms as shown in the legend. The “unclassified” in the species name refers to the sequences that could be attributed to more than one species with equal likelihoods. The isolated nodes represent the absence of co-occurrence among the species

We used the abundance data to understand the relationship among the microbial species detected in the samples. We computed the Bray-Curtis similarity among the samples (see “Methods”) to create two microbial community networks, one for each feeding type (Fig. 3). We conducted a permutation test to check whether our computed microbial community network could have been generated just by chance. We ran a permutation test against 1000 randomly permuted networks with the same set of nodes (preserving the network degree). The *p* value of 0.0465 for FF networks, and 0.0008 for BF network was obtained (showing that the networks were not just by chance).

The FF microbial community network is ~1.5 times denser than the corresponding BF network. This can be seen as a direct implication of the higher diversity observed in FF infant microbiota and leads to an intricate mutual dependence of microbes on one another. Furthermore, among the three *Bifidobacterium* species detected in our analysis, *B. breve* and *B. bifidum* were detected mostly in BF infants, whereas *B. longum* and *R. gnavus* were detected in FF samples (Fig. 2b). This also shows that the network at the phylum may look alike but at lower level (here, species level), the two systems (BF and FF) can be differentiated. The core of the BF network consisting of the largest connected component (11 nodes) was retained in the FF network but extended with several other co-occurrence relationships observed (Fig. 3).

### Relation between microbes and human systems

**Fig. 4:**
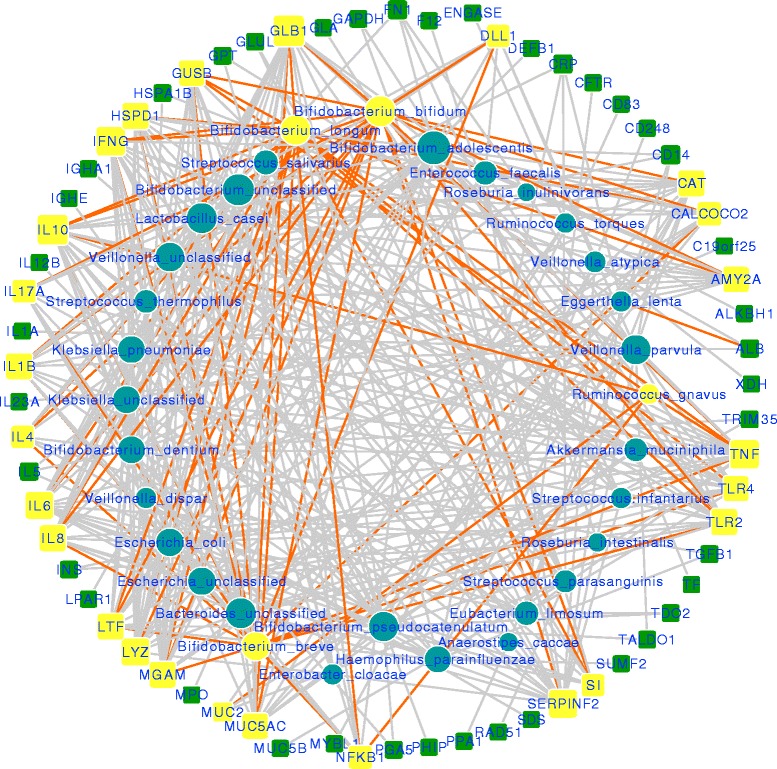
The microbial species and human gene relationships mined from literature in the form of a bipartite network. The size of nodes indicate the degree of vertices, i.e., the number of vertices connected to it. The *yellow circles* represent differentially abundant microbes, and *yellow squares* are the host genes that showed a literature-based relationship with these microbial species. The *orange-colored edges* connect differentially abundant microbes with their related host genes. A network restricted only to selected vertices in the graph is in Additional file 1

**Fig. 5:**
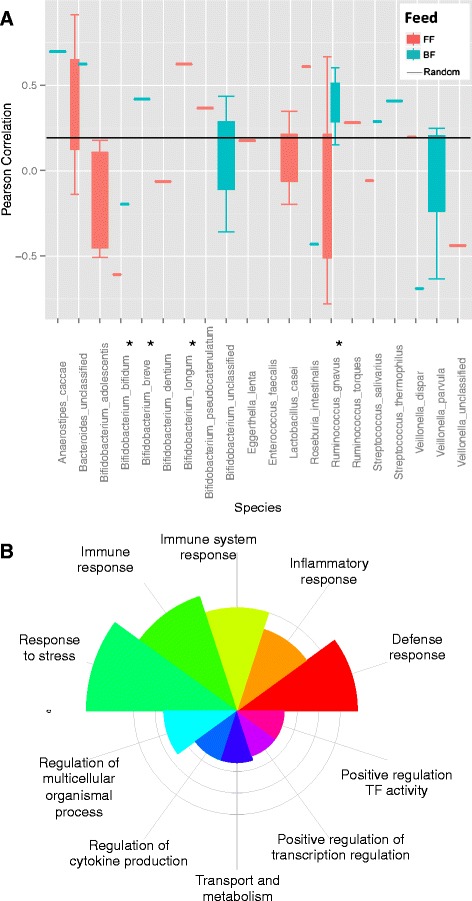
**a** Pearson correlation between the microbial abundance and the expression levels of associated human genes obtained via text mining and also found to be differentially expressed. The relationship with human genes for each microbial species is in Fig. 4. The microbial species marked with *asterisk* (*) are differentially abundant. The *black horizontal line* is the mean of absolute Pearson correlation coefficient between randomly generated pairs of genes and microbes. The missing sample (BF or FF) had an NA as correlation values due to zero abundance or zero standard deviation in any of the random variables. **b** The top GO terms (Biological Process) for human genes related to microbial species. The width of sectors represents the number of associated terms in the corresponding categories, and the radius indicates the number of genes annotated with corresponding terms

We extracted the relations of microbes with the human genes from bibliographic knowledgebase (see “Methods”). These genes do not necessarily represent a physical interaction with microbe or its biomolecules but rather a dependence relationship in either direction (gray edges in Fig. 4). Looking at the related genes (square nodes) for differentially abundant microbes (a network specific to differentially abundant species is available in Additional file 1) revealed that they are mostly related to the host genes (Fig. 4, yellow nodes in circle and squares) that already have a higher degree. To further verify these relationships, we analyzed which host genes have been found to be in a dependence relation with the corresponding microbial species via a bivariate analysis. For this, we used the pairs of microbes and the host genes related to these microbes as shown in Fig. 4. To illustrate, if a microbe M is related to host genes X, Y, and Z; then, we compute the correlation for abundance of M and expression level of X, Y, and Z along the samples. Thus, we get three correlation values for microbe M, i.e., M~X, M~Y, and M~Z. This was carried out for all the microbes and their corresponding genes (related to these microbes). The Pearson correlation coefficients were computed between microbial abundance and the expression levels of corresponding DE genes (a set of *n* correlation coefficients for a species enriched with *n* genes) showed a higher correlation (*p* value ≤0.05) for *B. bifidum* and *R. gnavus*. Figure 5a shows the boxplot for the correlations between each microbial species, paired with its corresponding genes extracted from literature. The results cannot be computed in terms of *p* values as the two other differentially abundant microbes (*B. breve* and *B. longum*) were not detected in both types of sample. We compared these correlation coefficients against the mean of absolute correlation between 1000 randomly permuted microbial species and host gene pairs, which was found to be <0.2. Furthermore, we also generated random pairs of genes and microbes and computed similar correlations (abundance~expression level) to check if the correlations for actual relationships are better than these random pairs. We compared these correlation coefficients against the mean of absolute correlation between 1000 randomly permuted microbial species and host gene pairs, which was found to be <0.2 (indicated by the black line in Fig. 5a). This approach also served as a validation for our literature-mining pipeline to find genes related to microbial species.

A functional enrichment analysis with GO terms (Biological Process) of the genes related to differentially abundant species indicated their role in the immune system (Fig. 5b; details in Additional file 2). Similar enrichment analysis for the genes related to all the species was found to be rich with immune system, metabolic- and transport-related terms (Fig. 5b). A pathway enrichment analysis based on Reactome [30] gave a similar result with several metabolic pathways, e.g., carbohydrate digestion, interleukin processing, and antigen processing.

### Differences at transcriptomic level in host

The analysis of the gene expression data obtained from the epithelial cells in fecal samples of the infants (see “Methods”) distinguishes the two feeding types at the transcription level (see Additional file 1). The functional enrichment analysis of the 477 differentially expressed genes revealed a significant abundance of immune system-related activities (Fig. 5a). We extended our analysis to obtain two co-expression networks of genes under each feeding condition. As a higher number of array probes provided overexpression signals in the transcriptomic data for BF infants, the BF gene co-expression networks (see “Methods” section) were ~2.1 denser than the FF network at the level of gene co-expression unlike our finding at microbial community level (see “The microbiota” section).

### The feeding mode microbiome host system

**Fig. 6:**
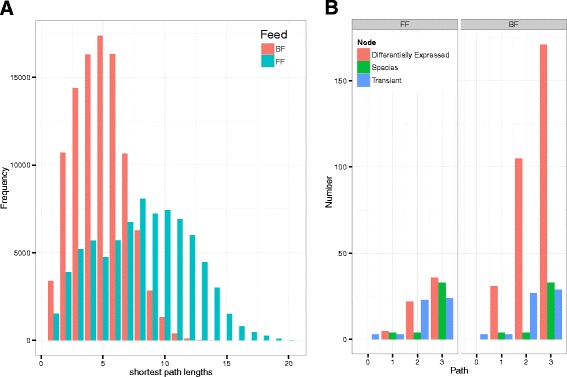
**a** Plot showing the distribution of shortest path lengths of a host gene-microbe network under FF and BF conditions. The left skewed distribution of BF networks with smaller diameter (14) shows the small-world properties of the network and a higher robustness (against perturbation) compared to the right skewed FF network with a diameter of 20. **b** The plot represents the number of nodes that can be reached (Y-axis) after traversing through certain path lengths, (here *1*, *2*, and *3* along X-axis). The node type referred here are the “DE genes” (differentially expressed genes) from gene expression data, “Species” are the microbes, and “Transient” nodes are the genes mined from literature that were found to share relationship with microbial species. The corresponding networks are available as a network file in Additional file 1 and Additional file 3 (can be opened in cytoscape) and as figures in Additional file 1

Combining the outcomes from the abovementioned results, we could layout the dependence of the microbiota and human system on feeding mode together with the relationship between the microbiota and the human system (see Additional file 1: Figures 0.10 and 0.11 and Additional file 3 (network SIF files)). The differences between the two feeding modes could be observed at this level (Fig. 6). Analyzing the topology of the two networks showed a higher diameter of 20 for the FF than for BF network (14). Furthermore, the BF network showed a considerably higher density and shorter average paths lengths than the FF network (Fig. 6a). These features indicate a greater degree of small-world properties in a BF network and hence robustness against perturbations [31]. To extend our analysis further, we measured the reachability of the nodes starting from a random node in the graph traversing a fixed path length (Fig. 6b). This allows us to measure connectivity in the networks. The results showed that within the denser BF network there are more dependencies (interactions) among the genes than the FF network. The two dense clusters of nodes visible in the BF network (see Additional file 1: Figure 0.10) supports the connectivity analysis done via Fig. 6b.

## Discussion

The main focus of this study was to complement the work of Schwartz et al. with a systems level analysis for understanding the colonization of infant gut as a function of feeding mode and its interaction between the microbiota and the human system. The feeding mode seemed to directly affect the microbiota as well as the host system. We complemented the approach of Schwartz et al. in three directions: (1) mining the relationships between the microbiota and the host genes from literature and their validation with the gene expression and microbial abundance data; (2) a differential abundance analysis of the microbiota at species level; and (3) combining these findings at a systems biology level depicting the overall relations between the feeding mode, the microbiota, and host biomolecular activities.

Feeding mode has been shown to affect the microbiota composition as well as gene expression in infants [23, 14, 21]. The presence of *Bacteroides* in BF samples was unusual, but it can be explained, as recent findings have shown that *Bacteroides* colonize infant gut with time [32, 33]. The samples we used were from 12-week-old infants; this likely explains the presence of *Bacteroides* in BF samples. Furthermore, it has also been shown that certain *Bacteroides* species can use the sugar in human milk via mucosal layers [34]. It has also been shown that the formula-fed infants had increased richness of species compared to the breast-fed infants [21, 35]. Our findings confirmed that formula-feeding increases the microbial diversity. This finding was in line with existing knowledge [19–22]. Nevertheless, the core of the co-occurrence microbial network was retained. In terms of microbial enrichment, we showed that *B. longum* was abundant in the FF infants. *B. longum* is one of the widely used probiotic in formula food [36]. The genes associated with differentially abundant microbes were highly involved in immunological activities depicting the role of these microbes in the functioning of the human system.

The picture was different at the level of human genes. The gene network among the BF sample was found to have a higher number of interactions (~2.1 times the FF network). Thus, the phenotypic outcomes observed in the host system are affected by feeding conditions. The BF samples do not exhibit a great diversity in the microbiota network [23]. The higher transcriptomic activities as inferred from a denser co-expression networks in BF infants indicates the presence of bioactive compounds in the breast milk [37]. These compounds can activate numerous pathways compared to formula [38]. The presence of genes related to metabolism in our co-expression network extends the effect of feeding mode to these activities together with the immune system [39, 40]. The difference at metabolomic level has been shown before by Martin et al. [41].

The conservation of the core of microbial co-occurrence networks and the differentially abundant microbes being associated with genes that already have higher degree, leads to the question of the true functional importance of diversity in the microbiota. However, a core microbiota is undoubtedly required for optimal health [42] and it has also been observed in our study, where the core of the community network was retained under both BF and FF conditions. At the level of network biology, the gain in microbial diversity was not found to add many new relationships/effects to the host system (genes) but rather presented alternative paths to the existing ones in the network for BF infants. However, the role of bioactive components of breast milk played a prominent role in activity at the host (human) level. As observed in the overall microbe-host gene system (Fig. 6b), the sharp increase in reachability of differentially expressed genes in BF infants is leveraged by the bioactive compounds in breast milk. The difference in the reachability of transient human genes (linked to microbial species) remained more consistent since new microbes had indifferent relationships to human genes. Nevertheless, they might provide alternate ways to affect the microbiota-host relationships.

Our findings based on small but unbiased samples leveraged by robust statistical checks suggested that the development of human immune system during infancy should not be seen as an effect of the diet or the microbiota at individual level, rather these factors should be studied together in a system level approach. Our results indicated how feeding affected the expression pattern of genes as well as the microbial community and how these two factors together impact the host system (infants).

## Conclusions

We used a network model with multiple components (microbial species and human genes) to study the effects of feeding conditions in infants on the microbiota and on the overall human system and its role in host immune system. The components included the microbial system and the host genes together with the interplay between these components. The analysis on the microbiota revealed a feeding mode-dependent difference in terms of diversity of the microbiota as well as of the network describing the interactions among them. The comparison of the results based on literature and data showed the validity of our findings. The network among the differentially expressed genes, computed via co-expression, was found to be much denser in the case of BF infants than FF infants, depicting higher biomolecular cross talk depending on the feeding mode. Many of the edges in the gene network for BF and FF samples could be mapped onto immune, signaling, or metabolic pathways. Mapping the relations between microbes and human genes was another major advancement achieved during the study. We complemented and extended the work of Schwartz et al. by detecting the differences in the microbiota based on feeding mode. The study of the microbiota and the host system together helped us to study a meta-system consisting of the host with the fellow microbes residing within the host rather than individual organism systems. Our results revealed that the integration of -omics data from the host and the microbiota with existing knowledge (in this case, literature) could yield useful insights into the system or rather the meta-system.

## Methods

### Data

We used (1) metagenomic and (2) transcriptomic data [14, 43] from a previous study by Schwartz et al. The metagenomic data was obtained from EBI Short Read Archive (SRA) accession number ERP001038. It consisted of microbial DNA profiles in full term 3-month-old infants obtained from the fecal samples via metagenomic pyrosequencing. The transcriptomic data in the form of gene expression profiles came from NCBI Gene Expression Omnibus (GEO) accession GSE31075. The mRNA measurements were performed on intact sloughed epithelial cells from fecal samples. Both types of data were taken from the same set of infants categorized into the two exclusive feeding conditions (BF and FF).

### Methods

Our approach aimed to use the metagenomic and transcriptomic data integratively in order to infer the relation between the microbiota, the host (human) system, and the feeding mode.

### Expression data analysis

The limma package [44] from Bioconductor was used to identify genes that were differentially expressed between feeding types. Limma uses an empirical Bayes method to test the differential expression of genes [45]. A *p* value cutoff of 0.05 (after multiple testing correction based on Benjamini-Hochberg method [46]) and a log fold change ≥2 were used to select the differentially expressed genes, resulting in 477 genes that were differentially expressed under the two feeding conditions.

### Metagenomic data analysis

We analyzed the metagenome data in the form of sequence reads in order to obtain the enrichment of microbes in the samples. We used Bowtie 2 [29] to align the reads to the microbial genome database. Then, we performed RAST analysis [47] to produce the microbial and functional annotation that identified the biomolecular functions of the sequences detected in the metagenomic data. We also computed the species abundance in the metagenomic sample using MetaPhlAn [28]. The data were then filtered to the species level to obtain the abundances of species in the metagenomic samples. A limma analysis [45] was used to detect the differential abundance of the species in the samples (for details, see [48]).

### Inferring microbial community network

Inferring microbial community network is a major challenge due to ambiguities about the operational taxonomic unit (OTU) definition and corresponding variation in quantification data based on OTUs. However, we define “microbial species” as the OTU in our investigation for simplicity and in order to follow the standards in the literature. The abundance table (see Additional file 4) computed for the metagenome from the samples showed many entries equal to zero. We adopted the Bray-Curtis similarity to obtain the pairwise distances between the microbial species based on their corresponding abundances across the samples. In this way, we infer the relationships between pairs of organisms, based on their co-occurrence patterns. We used Bray-Curtis similarity because (1) it is insensitive to sparse count data (i.e., the occurrence of zeroes in the count data because of the absence or below detection level abundance of a microbe [49]) and (2) correlation coefficients cannot serve the purpose due to the small sample size (six BF and six FF). Similarities with mean scores less than least 2 standard deviations above 0 were filtered out to disallow large variance in the similarity matrix, thereby removing spurious edges in the network (i.e., a kind of sampling noise). This matrix was used to infer the relations among the microbial species.

### Extracting microbe-gene relations from text

The knowledge of potential bipartite relations between the microbes and the human genes/proteins is important to establish effects of feeding modes and microbes on the human mechanisms at system level. To extract these relations, we performed text mining on MEDLINE abstracts [50–52]. We identified the abstracts containing the co-occurrence of human genes in context with the microbial species under investigation. This list went through a filter to include only the text that had relationship terms like “activates,” “interacts with,” “depends on,” etc. Thus, a set of genes/proteins for each microbial species was created. This can be interpreted as microbial species enriched in relationships with certain human genes. To identify statistically significant genes/protein for each microbial species, we did a Fisher’s exact test on this enrichment. The relationships with *p* values less than 0.05 were accepted for further analysis (see Additional file 5). The approach used the statistical test score to establish relations between a microbe and genes retrieving only significant and specific relations from the text and minimizing noise.

### Computing the gene-gene co-expression network

Based on the set of differentially expressed genes extracted from the expression data, together with those mined from literature, the gene-gene relationships were computed. A co-expression gene network based on the Pearson correlation coefficient was created based on these data for two different conditions (breast-fed and formula-fed samples). The cutoff for the correlation was set to 0.8. For simplicity, unweighted networks were constructed. The networks were joined with the microbial community networks and the results from text mining procedure to get an overall picture.

### Functional analysis

We did a hyper-geometric test on the genes in the data from different sources (expression, literature) to understand the biological roles of the human genes involved by means of a functional enrichment of these gene sets. A detailed analysis of this functional enrichment was done at individual and combinatorial level. The differentially expressed genes and text mining genes underwent functional enrichment with GO Biological Process terms [53] and Reactome pathways [30].

## Additional files

Additional file 1:
**Supporting supplementary figures for data analysis.** (PDF 2,238 kb) Additional file 2:
**GO enrichment tables for human genes involved in the system.** (CSV 79 kb) Additional file 3:
**Network files for the two feeding conditions.** (PDF 134 kb) Additional file 4:
**Microbial abundance tables.** (CSV 14 kb) Additional file 5:
**Text-mining-based relations: microbial species and human genes.** (CSV 14 kb)
