# Supplementary material for: The role of breast-feeding in infant immune system: a systems perspective on the intestinal microbiome
Source: Microbiome. 2015 Sep 24;3:41. doi: 10.1186/s40168-015-0104-7 (PMC4581423; doi:10.1186/s40168-015-0104-7)
Supplement: Additional file 3: — Network files for the two feeding conditions. (PDF 134 kb) [file 40168_2015_104_MOESM3_ESM.pdf]

Breast Fed Network

Source	Type	Target
XDH	none	Veillonella_atypica
TRIM35	none	Bifidobacterium_dentium
TRIM35	none	Bifidobacterium_pseudocatenulatum
TNF	none	Akkermansia_muciniphila
TNF	none	Ruminococcus_gnavus
TNF	none	Bifidobacterium_dentium
TNF	none	Klebsiella_unclassified
TNF	none	Klebsiella_pneumoniae
TNF	none	Bifidobacterium_breve
TNF	none	Haemophilus_parainfluenzae
TNF	none	Streptococcus_thermophilus
TNF	none	Veillonella_unclassified
TNF	none	Veillonella_parvula
TNF	none	Lactobacillus_casei
TNF	none	Eubacterium_limosum
TNF	none	Bifidobacterium_unclassified
TNF	none	Bifidobacterium_longum
TNF	none	Bifidobacterium_bifidum
TNF	none	Bifidobacterium_adolescentis
TNF	none	Bacteroides_unclassified
TNF	none	Escherichia_unclassified
TNF	none	Escherichia_coli
TLR4	none	Klebsiella_unclassified
TLR4	none	Klebsiella_pneumoniae
TLR4	none	Bifidobacterium_breve
TLR4	none	Veillonella_unclassified
TLR4	none	Veillonella_parvula
TLR4	none	Lactobacillus_casei
TLR4	none	Eubacterium_limosum
TLR4	none	Bifidobacterium_unclassified
TLR4	none	Bifidobacterium_longum
TLR4	none	Bifidobacterium_adolescentis
TLR4	none	Bacteroides_unclassified
TLR4	none	Escherichia_unclassified
TLR4	none	Escherichia_coli
TLR2	none	Klebsiella_unclassified
TLR2	none	Klebsiella_pneumoniae
TLR2	none	Bifidobacterium_breve
TLR2	none	Veillonella_unclassified
TLR2	none	Veillonella_parvula
TLR2	none	Lactobacillus_casei
TLR2	none	Bifidobacterium_unclassified
TLR2	none	Bifidobacterium_longum
TLR2	none	Bifidobacterium_bifidum
TLR2	none	Bifidobacterium_adolescentis
TLR2	none	Bifidobacterium_pseudocatenulatum
TLR2	none	Enterococcus_faecalis
TLR2	none	Bacteroides_unclassified
TLR2	none	Escherichia_unclassified

TLR2	none	Escherichia_coli
TGFB1	none	Bifidobacterium_dentium
TGFB1	none	Haemophilus_parainfluenzae
TF	none	Akkermansia_muciniphila
TDO2	none	Eubacterium_limosum
TDO2	none	Bifidobacterium_pseudocatenulatum
TALDO1	none	Veillonella_parvula
TALDO1	none	Bifidobacterium_pseudocatenulatum
SUMF2	none	Haemophilus_parainfluenzae
SI	none	Streptococcus_thermophilus
SI	none	Bifidobacterium_unclassified
SI	none	Streptococcus_salivarius
SI	none	Bifidobacterium_longum
SI	none	Bifidobacterium_bifidum
SI	none	Bifidobacterium_adolescentis
SI	none	Bacteroides_unclassified
SI	none	Escherichia_unclassified
SI	none	Escherichia_coli
SERPINF:	none	Streptococcus.infantarius
SERPINF:	none	Anaerostipes_caccae
SERPINF:	none	Veillonella_dispar
SERPINF:	none	Klebsiella_unclassified
SERPINF:	none	Klebsiella_pneumoniae
SERPINF:	none	Enterobacter_cloacae
SERPINF:	none	Veillonella_atypica
SERPINF:	none	Haemophilus_parainfluenzae
SERPINF:	none	Eggerthella_lenta
SERPINF:	none	Veillonella_unclassified
SERPINF:	none	Veillonella_parvula
SERPINF:	none	Lactobacillus_casei
SERPINF:	none	Bifidobacterium_unclassified
SERPINF:	none	Streptococcus_salivarius
SERPINF:	none	Bifidobacterium_bifidum
SERPINF:	none	Bifidobacterium_adolescentis
SERPINF:	none	Enterococcus_faecalis
SERPINF:	none	Bacteroides_unclassified
SDS	none	Veillonella_dispar
RAD51	none	Streptococcus_parasanguinis
PPA1	none	Anaerostipes_caccae
PPA1	none	PPA1
PPA1	none	SRSF10
PPA1	none	SPINT2
PHIP	none	Bifidobacterium_pseudocatenulatum
PGA5	none	Bifidobacterium_dentium
PGA5	none	Bifidobacterium_pseudocatenulatum
NFKB1	none	Ruminococcus_gnavus
NFKB1	none	Bifidobacterium_breve
NFKB1	none	Lactobacillus_casei
NFKB1	none	Bifidobacterium_unclassified
NFKB1	none	Streptococcus_salivarius

NFKB1	none	Bifidobacterium_longum
NFKB1	none	Bifidobacterium_adolescentis
NFKB1	none	Bacteroides_unclassified
NFKB1	none	Escherichia_unclassified
NFKB1	none	Escherichia_coli
MYBL1	none	Veillonella_atypica
MUC5B	none	Streptococcus_parasanguinis
MUC5AC	none	Akkermansia_muciniphila
MUC5AC	none	Bifidobacterium_dentium
MUC5AC	none	Bifidobacterium_breve
MUC5AC	none	Ruminococcus_torques
MUC5AC	none	Eggerthella_lenta
MUC5AC	none	Veillonella_unclassified
MUC5AC	none	Veillonella_parvula
MUC5AC	none	Bifidobacterium_unclassified
MUC5AC	none	Streptococcus_salivarius
MUC5AC	none	Bifidobacterium_longum
MUC5AC	none	Bifidobacterium_bifidum
MUC5AC	none	Bifidobacterium_adolescentis
MUC5AC	none	Bifidobacterium_pseudocatenulatum
MUC5AC	none	Bacteroides_unclassified
MUC2	none	Akkermansia_muciniphila
MUC2	none	Ruminococcus_gnavus
MUC2	none	Streptococcus_parasanguinis
MUC2	none	Ruminococcus_torques
MPO	none	Bifidobacterium_adolescentis
MGAM	none	Klebsiella_unclassified
MGAM	none	Klebsiella_pneumoniae
MGAM	none	Bifidobacterium_breve
MGAM	none	Streptococcus_thermophilus
MGAM	none	Veillonella_unclassified
MGAM	none	Veillonella_parvula
MGAM	none	Lactobacillus_casei
MGAM	none	Bifidobacterium_unclassified
MGAM	none	Streptococcus_salivarius
MGAM	none	Bifidobacterium_longum
MGAM	none	Bifidobacterium_bifidum
MGAM	none	Bifidobacterium_adolescentis
MGAM	none	Bacteroides_unclassified
MGAM	none	Escherichia_unclassified
MGAM	none	Escherichia_coli
LYZ	none	Veillonella_dispar
LYZ	none	Klebsiella_unclassified
LYZ	none	Klebsiella_pneumoniae
LYZ	none	Enterobacter_cloacae
LYZ	none	Streptococcus_thermophilus
LYZ	none	Veillonella_unclassified
LYZ	none	Veillonella_parvula
LYZ	none	Lactobacillus_casei
LYZ	none	Bifidobacterium_unclassified

LYZ	none	Streptococcus_salivarius
LYZ	none	Bifidobacterium_longum
LYZ	none	Bifidobacterium_bifidum
LYZ	none	Bifidobacterium_adolescentis
LYZ	none	Enterococcus_faecalis
LYZ	none	Bacteroides_unclassified
LYZ	none	Escherichia_unclassified
LYZ	none	Escherichia_coli
LTF	none	Bifidobacterium_dentium
LTF	none	Klebsiella_unclassified
LTF	none	Klebsiella_pneumoniae
LTF	none	Bifidobacterium_breve
LTF	none	Haemophilus_parainfluenzae
LTF	none	Streptococcus_thermophilus
LTF	none	Bifidobacterium_unclassified
LTF	none	Bifidobacterium_longum
LTF	none	Bifidobacterium_bifidum
LTF	none	Bifidobacterium_adolescentis
LTF	none	Bifidobacterium_pseudocatenulatum
LTF	none	Enterococcus_faecalis
LTF	none	Bacteroides_unclassified
LTF	none	Escherichia_unclassified
LTF	none	Escherichia_coli
LPAR1	none	Veillonella_dispar
INS	none	Roseburia_intestinalis
INS	none	Akkermansia_muciniphila
IL8	none	Streptococcus.infantarius
IL8	none	Klebsiella_unclassified
IL8	none	Klebsiella_pneumoniae
IL8	none	Bifidobacterium_breve
IL8	none	Haemophilus_parainfluenzae
IL8	none	Lactobacillus_casei
IL8	none	Bifidobacterium_unclassified
IL8	none	Streptococcus_salivarius
IL8	none	Bifidobacterium_longum
IL8	none	Bifidobacterium_bifidum
IL8	none	Bifidobacterium_adolescentis
IL8	none	Roseburia_inulinivorans
IL8	none	Bacteroides_unclassified
IL8	none	Escherichia_unclassified
IL8	none	Escherichia_coli
IL6	none	Roseburia_intestinalis
IL6	none	Akkermansia_muciniphila
IL6	none	Klebsiella_unclassified
IL6	none	Klebsiella_pneumoniae
IL6	none	Haemophilus_parainfluenzae
IL6	none	Veillonella_unclassified
IL6	none	Veillonella_parvula
IL6	none	Lactobacillus_casei
IL6	none	Eubacterium_limosum

IL6	none	Bifidobacterium_unclassified
IL6	none	Bifidobacterium_longum
IL6	none	Bifidobacterium_bifidum
IL6	none	Bifidobacterium_adolescentis
IL6	none	Bifidobacterium_pseudocatenulatum
IL6	none	Bacteroides_unclassified
IL6	none	Escherichia_unclassified
IL6	none	Escherichia_coli
IL5	none	Bifidobacterium_dentium
IL5	none	Bifidobacterium_adolescentis
IL5	none	Bifidobacterium_pseudocatenulatum
IL4	none	Bifidobacterium_dentium
IL4	none	Bifidobacterium_breve
IL4	none	Haemophilus_parainfluenzae
IL4	none	Lactobacillus_casei
IL4	none	Eubacterium_limosum
IL4	none	Bifidobacterium_unclassified
IL4	none	Bifidobacterium_longum
IL4	none	Bifidobacterium_bifidum
IL4	none	Bifidobacterium_adolescentis
IL23A	none	Veillonella_parvula
IL1B	none	Klebsiella_unclassified
IL1B	none	Klebsiella_pneumoniae
IL1B	none	Bifidobacterium_breve
IL1B	none	Haemophilus_parainfluenzae
IL1B	none	Veillonella_unclassified
IL1B	none	Veillonella_parvula
IL1B	none	Lactobacillus_casei
IL1B	none	Eubacterium_limosum
IL1B	none	Bifidobacterium_unclassified
IL1B	none	Bifidobacterium_longum
IL1B	none	Bifidobacterium_bifidum
IL1B	none	Bifidobacterium_adolescentis
IL1B	none	Bacteroides_unclassified
IL1B	none	Escherichia_unclassified
IL1B	none	Escherichia_coli
IL1A	none	Veillonella_parvula
IL17A	none	Akkermansia_muciniphila
IL17A	none	Klebsiella_unclassified
IL17A	none	Klebsiella_pneumoniae
IL17A	none	Bifidobacterium_breve
IL17A	none	Lactobacillus_casei
IL17A	none	Bifidobacterium_unclassified
IL17A	none	Bifidobacterium_bifidum
IL17A	none	Bacteroides_unclassified
IL17A	none	Escherichia_unclassified
IL17A	none	Escherichia_coli
IL12B	none	Bifidobacterium_pseudocatenulatum
IL10	none	Akkermansia_muciniphila
IL10	none	Ruminococcus_gnavus

IL10	none	Bifidobacterium_dentium
IL10	none	Klebsiella_unclassified
IL10	none	Klebsiella_pneumoniae
IL10	none	Bifidobacterium_breve
IL10	none	Haemophilus_parainfluenzae
IL10	none	Streptococcus_thermophilus
IL10	none	Veillonella_unclassified
IL10	none	Veillonella_parvula
IL10	none	Lactobacillus_casei
IL10	none	Eubacterium_limosum
IL10	none	Bifidobacterium_unclassified
IL10	none	Bifidobacterium_longum
IL10	none	Bifidobacterium_bifidum
IL10	none	Bifidobacterium_adolescentis
IL10	none	Bifidobacterium_pseudocatenulatum
IL10	none	Enterococcus_faecalis
IL10	none	Bacteroides_unclassified
IL10	none	Escherichia_unclassified
IL10	none	Escherichia_coli
IGHE	none	Bifidobacterium_pseudocatenulatum
IGHA1	none	Streptococcus_parasanguinis
IFNG	none	Akkermansia_muciniphila
IFNG	none	Bifidobacterium_dentium
IFNG	none	Klebsiella_unclassified
IFNG	none	Klebsiella_pneumoniae
IFNG	none	Bifidobacterium_breve
IFNG	none	Haemophilus_parainfluenzae
IFNG	none	Streptococcus_thermophilus
IFNG	none	Veillonella_unclassified
IFNG	none	Veillonella_parvula
IFNG	none	Lactobacillus_casei
IFNG	none	Eubacterium_limosum
IFNG	none	Bifidobacterium_unclassified
IFNG	none	Bifidobacterium_longum
IFNG	none	Bifidobacterium_bifidum
IFNG	none	Bifidobacterium_adolescentis
IFNG	none	Bifidobacterium_pseudocatenulatum
IFNG	none	Bacteroides_unclassified
IFNG	none	Escherichia_unclassified
IFNG	none	Escherichia_coli
HSPD1	none	Bifidobacterium_dentium
HSPD1	none	Streptococcus_parasanguinis
HSPD1	none	Klebsiella_unclassified
HSPD1	none	Klebsiella_pneumoniae
HSPD1	none	Enterobacter_cloacae
HSPD1	none	Bifidobacterium_breve
HSPD1	none	Streptococcus_thermophilus
HSPD1	none	Lactobacillus_casei
HSPD1	none	Bifidobacterium_unclassified
HSPD1	none	Streptococcus_salivarius

HSPD1	none	Bifidobacterium_longum
HSPD1	none	Bifidobacterium_adolescentis
HSPD1	none	Bifidobacterium_pseudocatenulatum
HSPD1	none	Enterococcus_faecalis
HSPD1	none	Bacteroides_unclassified
HSPA1B	none	Veillonella_dispar
HSPA1B	none	Veillonella_atypica
GUSB	none	Streptococcus.infantarius
GUSB	none	Ruminococcus_gnavus
GUSB	none	Bifidobacterium_dentium
GUSB	none	Enterobacter_cloacae
GUSB	none	Bifidobacterium_breve
GUSB	none	Veillonella_unclassified
GUSB	none	Lactobacillus_casei
GUSB	none	Bifidobacterium_unclassified
GUSB	none	Bifidobacterium_longum
GUSB	none	Bifidobacterium_bifidum
GUSB	none	Bifidobacterium_pseudocatenulatum
GUSB	none	Bacteroides_unclassified
GUSB	none	Escherichia_unclassified
GUSB	none	Escherichia_coli
GPT	none	Eggerthella_lenta
GPT	none	Bifidobacterium_pseudocatenulatum
GLUL	none	Streptococcus_parasanguinis
GLUL	none	Eubacterium_limosum
GLUL	none	GLUL
GLUL	none	TBX18
GLUL	none	KIAA1551
GLUL	none	09/01/14
GLUL	none	CEBPB
GLUL	none	U2AF1
GLUL	none	MGEA5
GLUL	none	SMAP2
GLUL	none	KU-MEL-3
GLB1	none	Streptococcus.infantarius
GLB1	none	Bifidobacterium_dentium
GLB1	none	Streptococcus_parasanguinis
GLB1	none	Klebsiella_unclassified
GLB1	none	Klebsiella_pneumoniae
GLB1	none	Enterobacter_cloacae
GLB1	none	Bifidobacterium_breve
GLB1	none	Haemophilus_parainfluenzae
GLB1	none	Streptococcus_thermophilus
GLB1	none	Veillonella_unclassified
GLB1	none	Veillonella_parvula
GLB1	none	Lactobacillus_casei
GLB1	none	Bifidobacterium_unclassified
GLB1	none	Streptococcus_salivarius
GLB1	none	Bifidobacterium_longum
GLB1	none	Bifidobacterium_bifidum

GLB1	none	Bifidobacterium_adolescentis
GLB1	none	Bifidobacterium_pseudocatenulatum
GLB1	none	Enterococcus_faecalis
GLB1	none	Bacteroides_unclassified
GLB1	none	Escherichia_unclassified
GLB1	none	Escherichia_coli
GLA	none	Streptococcus.infantarius
GAPDH	none	Akkermansia_muciniphila
GAPDH	none	Streptococcus_parasanguinis
GAPDH	none	GAPDH
GAPDH	none	SPATS2L
GAPDH	none	NDUFB8
GAPDH	none	EEF1A1
GAPDH	none	RBM22
GAPDH	none	CD63
GAPDH	none	SGK1
GAPDH	none	PMAIP1
FN1	none	Streptococcus.infantarius
FN1	none	Veillonella_unclassified
FN1	none	Veillonella_parvula
FN1	none	Bifidobacterium_adolescentis
F12	none	Roseburia_intestinalis
ENGASE	none	Ruminococcus_torques
DLL1	none	Bifidobacterium_breve
DLL1	none	Veillonella_atypica
DLL1	none	Veillonella_unclassified
DLL1	none	Veillonella_parvula
DLL1	none	Lactobacillus_casei
DLL1	none	Streptococcus_salivarius
DLL1	none	Bifidobacterium_bifidum
DLL1	none	Bifidobacterium_adolescentis
DLL1	none	Enterococcus_faecalis
DEFB1	none	Bifidobacterium_dentium
CRP	none	Streptococcus_parasanguinis
CRP	none	Haemophilus_parainfluenzae
CRP	none	Eggerthella_lenta
CRP	none	Veillonella_unclassified
CFTR	none	Eubacterium_limosum
CD83	none	Bifidobacterium_pseudocatenulatum
CD248	none	Haemophilus_parainfluenzae
CD14	none	Veillonella_unclassified
CD14	none	Veillonella_parvula
CD14	none	Eubacterium_limosum
CD14	none	Bifidobacterium_adolescentis
CAT	none	Anaerostipes_caccae
CAT	none	Bifidobacterium_dentium
CAT	none	Ruminococcus_torques
CAT	none	Haemophilus_parainfluenzae
CAT	none	Eggerthella_lenta
CAT	none	Veillonella_unclassified

CAT	none	Veillonella_parvula
CAT	none	Lactobacillus_casei
CAT	none	Bifidobacterium_unclassified
CAT	none	Streptococcus_salivarius
CAT	none	Bifidobacterium_bifidum
CAT	none	Bifidobacterium_adolescentis
CAT	none	Bifidobacterium_pseudocatenulatum
CAT	none	Enterococcus_faecalis
CAT	none	Bacteroides_unclassified
CALCOC(	none	Enterobacter_cloacae
CALCOC(	none	Bifidobacterium_breve
CALCOC(	none	Lactobacillus_casei
CALCOC(	none	Bifidobacterium_unclassified
CALCOC(	none	Bifidobacterium_longum
CALCOC(	none	Bifidobacterium_bifidum
CALCOC(	none	Bifidobacterium_adolescentis
CALCOC(	none	Enterococcus_faecalis
CALCOC(	none	Bacteroides_unclassified
CALCOC(	none	Escherichia_unclassified
CALCOC(	none	Escherichia_coli
C19orf25	none	Veillonella_dispar
AMY2A	none	Bifidobacterium_dentium
AMY2A	none	Klebsiella_unclassified
AMY2A	none	Klebsiella_pneumoniae
AMY2A	none	Veillonella_atypica
AMY2A	none	Streptococcus_thermophilus
AMY2A	none	Veillonella_unclassified
AMY2A	none	Lactobacillus_casei
AMY2A	none	Bifidobacterium_unclassified
AMY2A	none	Streptococcus_salivarius
AMY2A	none	Bifidobacterium_longum
AMY2A	none	Bifidobacterium_bifidum
AMY2A	none	Bifidobacterium_adolescentis
AMY2A	none	Enterococcus_faecalis
AMY2A	none	Roseburia_inulinivorans
ALKBH1	none	Ruminococcus_torques
ALB	none	Veillonella_atypica
ALB	none	Eggerthella_lenta
ALB	none	Veillonella_parvula
S100A16	none	S100A16
S100A16	none	GADD45A
S100A16	none	CHP1
S100A16	none	EIF4E2
S100A16	none	ISG20
S100A16	none	PPARG
S100A16	none	PTS
S100A16	none	CALM2
S100A16	none	TNFRSF21
S100A16	none	BAIAP2L1
S100A16	none	TINAG

S100A16	none	CKS1B
S100A16	none	PSMB3
S100A16	none	TCEB1
S100A16	none	ZFAND5
S100A16	none	NSL1
S100A16	none	CD164
S100A16	none	GNG5
S100A16	none	CLCA4
S100A16	none	CAPZA2
S100A16	none	ZCCHC24
S100A16	none	PRSS3
S100A16	none	RHEB
S100A16	none	RPS27L
S100A16	none	SPCS3
S100A16	none	PSMB8
S100A16	none	BUD31
S100A16	none	C15orf48
S100A16	none	SPATS2L
S100A16	none	POLR2F
S100A16	none	PAK1
S100A16	none	DPY30
S100A16	none	B4GALT5
S100A16	none	CD63
S100A16	none	ARPC5
S100A16	none	ZFAND2A
S100A16	none	MMADHC
TNFAIP6	none	TNFAIP6
TNFAIP6	none	PDE4B
TNFAIP6	none	LAPTM5
TNFAIP6	none	BRIP1
TNFAIP6	none	PGS1
TNFAIP6	none	GOSR1
TNFAIP6	none	RGS2
TNFAIP6	none	GLCCI1
PEPD	none	PEPD
PEPD	none	LRTM1
PEPD	none	DNAJB1
PEPD	none	ACTR10
PEPD	none	BAG3
LRTM1	none	LRTM1
LRTM1	none	DNAJB1
LRTM1	none	FOXJ3
LRTM1	none	BAG3
PDE4B	none	PDE4B
PDE4B	none	GOSR1
SS18L2	none	SS18L2
SS18L2	none	CEBPG
SS18L2	none	POLR3GL
SS18L2	none	VAPA
SS18L2	none	LXN
SS18L2	none	ZSWIM2
SS18L2	none	SETD9

SS18L2	none	CEACAM6
SS18L2	none	ARL6IP5
SS18L2	none	WAC-AS1
SS18L2	none	DPY30
SS18L2	none	CD63
SS18L2	none	VPS26B
CD24	none	CD24
CD24	none	FGD6
CD24	none	ASNSD1
CD24	none	UBXN2B
SRP14	none	SRP14
SRP14	none	FKBP3
SRP14	none	CEBPG
SRP14	none	FXYD3
SRP14	none	ORMDL1
SRP14	none	PTS
SRP14	none	PPP2CB
SRP14	none	POLR3GL
SRP14	none	TINAG
SRP14	none	CKS1B
SRP14	none	VAPA
SRP14	none	LXN
SRP14	none	CEACAM6
SRP14	none	RPS27L
SRP14	none	SMAD4
SRP14	none	RPL18
SRP14	none	ISX
SRP14	none	ANAPC16
SRP14	none	HPGD
SRP14	none	CD63
SRP14	none	VPS26B
SRP14	none	SMIM14
SRP14	none	ACTR10
SRP14	none	RAB11A
SRP14	none	SHC1
SRP14	none	HIST1H2AC
FKBP3	none	FKBP3
FKBP3	none	ORMDL1
FKBP3	none	ATP5C1
FKBP3	none	VAPA
FKBP3	none	AGR3
FKBP3	none	GOLGA4
FKBP3	none	LXN
FKBP3	none	SPINT2
FKBP3	none	HPGD
SDCBP	none	SDCBP
SDCBP	none	TSPO
SDCBP	none	HNRNPH3
SDCBP	none	U2AF1
SDCBP	none	ITM2B
SDCBP	none	ACP1
SDCBP	none	GAPDH

SDCBP	none	GTF2B
SDCBP	none	RBM22
SDCBP	none	CD63
SDCBP	none	PMAIP1
GADD45A	none	GADD45A
GADD45A	none	CEBPG
GADD45A	none	PPARG
GADD45A	none	PTS
GADD45A	none	TINAG
GADD45A	none	CEACAM6
GADD45A	none	RHEB
GADD45A	none	VPS26B
GADD45A	none	ZFAND2A
GADD45A	none	ABCB1
GADD45A	none	RAB1A
RPL7	none	RPL7
RPL7	none	ITM2B
RPL7	none	RPS9
RPL7	none	MGST3
RPL7	none	TARDBP
RPL7	none	GTF2B
RPL7	none	NDUFB8
RPL7	none	COG1
RPL7	none	SGK1
CHP1	none	CHP1
CHP1	none	EIF4E2
CHP1	none	UQCRH
CHP1	none	ADI1
CHP1	none	ANAPC11
CHP1	none	CALM2
CHP1	none	PPP2CB
CHP1	none	CKS1B
CHP1	none	TSPO
CHP1	none	TCEB1
CHP1	none	NSL1
CHP1	none	GNG5
CHP1	none	ITM2B
CHP1	none	PRSS3
CHP1	none	RPS9
CHP1	none	RPS27L
CHP1	none	GAPDH
CHP1	none	SPATS2L
CHP1	none	NDUFB8
CHP1	none	B4GALT5
CHP1	none	CD63
CHP1	none	SGK1
CHP1	none	HRCT1
CHP1	none	MMADHC
CHCHD2	none	CHCHD2
CHCHD2	none	EIF4E2
CHCHD2	none	TCEB1
CHCHD2	none	OSER1

CHCHD2	none	PRSS3	
CHCHD2	none	BUD31	
CHCHD2	none	SERPINB1	
CHCHD2	none	C15orf48	
CHCHD2	none	AREG	
EIF4E2	none	EIF4E2	
EIF4E2	none	PPARG	
EIF4E2	none	PTS	
EIF4E2	none	CALM2	
EIF4E2	none	BAIAP2L1	
EIF4E2	none	TINAG	
EIF4E2	none	CKS1B	
EIF4E2	none	TCEB1	
EIF4E2	none	GNG5	
EIF4E2	none	PRSS3	
EIF4E2	none	RHEB	
EIF4E2	none	SPCS3	
EIF4E2	none	BUD31	
EIF4E2	none	C15orf48	
EIF4E2	none	SPATS2L	
EIF4E2	none	POLR2F	
EIF4E2	none	B4GALT5	
EIF4E2	none	CD63	
EIF4E2	none	MMADHC	
TBX18	none	TBX18	
TBX18	none	KIAA1551	
TBX18	none		09/01/14
LAMC2	none	LAMC2	
LAMC2	none	TSPO	
LAMC2	none	TICAM1	
UQCRH	none	UQCRH	
UQCRH	none	TCEB1	
UQCRH	none	PRSS3	
UQCRH	none	BUD31	
UQCRH	none	GAPDH	
UQCRH	none	NDUFB8	
UQCRH	none	EEF1A1	
UQCRH	none	RBM22	
UQCRH	none	CD63	
UQCRH	none	SGK1	
CEBPG	none	CEBPG	
CEBPG	none	FXYD3	
CEBPG	none	PTS	
CEBPG	none	POLR3GL	
CEBPG	none	TNFRSF21	
CEBPG	none	TINAG	
CEBPG	none	CKS1B	
CEBPG	none	VAPA	
CEBPG	none	LXN	
CEBPG	none	ZSWIM2	
CEBPG	none	SETD9	
CEBPG	none	CEACAM6	

CEBPG	none	ARL6IP5
CEBPG	none	WAC-AS1
CEBPG	none	HPGD
CEBPG	none	DPY30
CEBPG	none	CD63
CEBPG	none	VPS26B
CEBPG	none	SHC1
DNAJB1	none	DNAJB1
DNAJB1	none	ACTN4
FXYD3	none	FXYD3
FXYD3	none	ADI1
FXYD3	none	ANAPC11
FXYD3	none	PTS
FXYD3	none	CALM2
FXYD3	none	PPP2CB
FXYD3	none	POLR3GL
FXYD3	none	TNFRSF21
FXYD3	none	TINAG
FXYD3	none	CKS1B
FXYD3	none	VAPA
FXYD3	none	NSL1
FXYD3	none	GNG5
FXYD3	none	LXN
FXYD3	none	CEACAM6
FXYD3	none	CMAS
FXYD3	none	RHEB
FXYD3	none	RPS27L
FXYD3	none	SMAD4
FXYD3	none	RPL18
FXYD3	none	ISX
FXYD3	none	ANAPC16
FXYD3	none	DPY30
FXYD3	none	CD63
FXYD3	none	ARPC5
FXYD3	none	SMIM14
FXYD3	none	ACTR10
FXYD3	none	RAB11A
FXYD3	none	SHC1
FXYD3	none	HRCT1
FXYD3	none	HIST1H2AC
FXYD3	none	MMADHC
BLOC1S1	none	BLOC1S1
BLOC1S1	none	MIER3
BLOC1S1	none	JTB
BLOC1S1	none	CMAS
BLOC1S1	none	CTGF
BLOC1S1	none	TARDBP
BLOC1S1	none	ISX
BLOC1S1	none	PBLD
BLOC1S1	none	DDX39B
BLOC1S1	none	CAMLG
BLOC1S1	none	SMIM14

BLOC1S1	none	ACTR10
BLOC1S1	none	HECA
ISG20	none	ISG20
ISG20	none	ZFAND5
ISG20	none	OSER1
ISG20	none	SPCS3
ISG20	none	PAK1
ISG20	none	AREG
NDUFB4	none	NDUFB4
NDUFB4	none	AGR3
NDUFB4	none	PPA1
NDUFB4	none	SRSF10
NDUFB4	none	SPINT2
ADCY4	none	ADCY4
ADCY4	none	LOC375295
ADCY4	none	LAPTM5
ADCY4	none	BRIP1
ADCY4	none	PGS1
PPP1CA	none	PPP1CA
PPP1CA	none	ORMDL1
PPP1CA	none	SEC61B
PPP1CA	none	VAPA
PPP1CA	none	PSMB3
PPP1CA	none	CD164
PPP1CA	none	CLCA4
PPP1CA	none	CAPZA2
PPP1CA	none	OAZ2
PPP1CA	none	RHEB
PPP1CA	none	SPCS3
PPP1CA	none	PSMB8
PPP1CA	none	RPL18
PPP1CA	none	NT5C3A
PPP1CA	none	SMIM20
PPP1CA	none	CTSS
PPP1CA	none	HPGD
PPP1CA	none	B4GALT5
PPP1CA	none	CBX3
PPP1CA	none	ARPC5
PPP1CA	none	SMIM14
PPP1CA	none	ACTR10
PPP1CA	none	RAB11A
PPP1CA	none	SHC1
PPP1CA	none	HIST1H2AC
PPP1CA	none	MMADHC
TMED4	none	TMED4
TMED4	none	CD164
TMED4	none	DHRS7
TMED4	none	IFI27L1
PPARG	none	PPARG
PPARG	none	PTS
PPARG	none	CALM2
PPARG	none	BAIAP2L1

PPARG	none	TINAG
PPARG	none	CEACAM6
PPARG	none	RHEB
PPARG	none	TMED9
PPARG	none	C15orf48
PPARG	none	POLR2F
PPARG	none	RAB1A
ORMDL1	none	ORMDL1
ORMDL1	none	VAPA
ORMDL1	none	C6orf211
ORMDL1	none	AGR3
ORMDL1	none	LXN
ORMDL1	none	RPL18
ORMDL1	none	ANAPC16
ORMDL1	none	HPGD
ORMDL1	none	SMIM14
ORMDL1	none	ACTR10
ORMDL1	none	RAB11A
ORMDL1	none	SHC1
ORMDL1	none	HIST1H2AC
FYN	none	FYN
FYN	none	RBPJ
C9orf72	none	C9orf72
C9orf72	none	PLOD2
C9orf72	none	SNRNP70
C9orf72	none	RBPJ
C9orf72	none	COG1
PLOD2	none	PLOD2
PLOD2	none	HNRNPH3
PLOD2	none	RBPJ
ATP5C1	none	ATP5C1
ATP5C1	none	TXNDC11
ATP5C1	none	AGR3
ATP5C1	none	PPA1
ATP5C1	none	SPINT2
ADI1	none	ADI1
ADI1	none	ANAPC11
ADI1	none	PTS
ADI1	none	PPP2CB
ADI1	none	CKS1B
ADI1	none	NSL1
ADI1	none	GNG5
ADI1	none	CLCA4
ADI1	none	JTB
ADI1	none	ITM2B
ADI1	none	CMAS
ADI1	none	RPS9
ADI1	none	MUTYH
ADI1	none	RPS27L
ADI1	none	MGST3
ADI1	none	PSMB8
ADI1	none	TARDBP

ADI1	none	RPL18
ADI1	none	NT5C3A
ADI1	none	SMIM20
ADI1	none	IL10RB
ADI1	none	SPATS2L
ADI1	none	GTF2B
ADI1	none	NDUFB8
ADI1	none	ANAPC16
ADI1	none	B4GALT5
ADI1	none	CBX3
ADI1	none	CD63
ADI1	none	ARPC5
ADI1	none	SMIM14
ADI1	none	ACTR10
ADI1	none	RAB11A
ADI1	none	SGK1
ADI1	none	SHC1
ADI1	none	HRCT1
ADI1	none	HIST1H2AC
ADI1	none	MMADHC
KIAA1551	none	KIAA1551
KIAA1551	none	LOC375295
KIAA1551	none	PITPNA
KIAA1551	none	09/01/14
KIAA1551	none	CEBPB
KIAA1551	none	U2AF1
KIAA1551	none	MGEA5
KIAA1551	none	ANXA3
KIAA1551	none	CCNL1
KIAA1551	none	BRIP1
KIAA1551	none	CD55
SEC61B	none	SEC61B
SEC61B	none	BAIAP2L1
SEC61B	none	CD164
SEC61B	none	POLR2F
SEC61B	none	HPGD
SEC61B	none	RAB9A
SEC61B	none	DHRS7
LOC3752	none	LOC375295
LOC3752	none	LAPTM5
LOC3752	none	BRIP1
TDP2	none	TDP2
TDP2	none	GOLGA4
TDP2	none	SMAD4
TDP2	none	RAB1A
ANAPC11	none	ANAPC11
ANAPC11	none	PTS
ANAPC11	none	CALM2
ANAPC11	none	PPP2CB
ANAPC11	none	CKS1B
ANAPC11	none	NSL1
ANAPC11	none	GNG5

ANAPC11	none	CLCA4
ANAPC11	none	JTB
ANAPC11	none	ITM2B
ANAPC11	none	CMAS
ANAPC11	none	RPS9
ANAPC11	none	RPS27L
ANAPC11	none	MGST3
ANAPC11	none	TARDBP
ANAPC11	none	NDUFB8
ANAPC11	none	ANAPC16
ANAPC11	none	B4GALT5
ANAPC11	none	CBX3
ANAPC11	none	CD63
ANAPC11	none	ARPC5
ANAPC11	none	CAMLG
ANAPC11	none	SMIM14
ANAPC11	none	ACTR10
ANAPC11	none	SHC1
ANAPC11	none	HRCT1
ANAPC11	none	MMADHC
GPSM3	none	GPSM3
GPSM3	none	PLEKHO1
GPSM3	none	GLCCI1
PTS	none	PTS
PTS	none	CALM2
PTS	none	PPP2CB
PTS	none	TNFRSF21
PTS	none	TINAG
PTS	none	CKS1B
PTS	none	VAPA
PTS	none	NSL1
PTS	none	CD164
PTS	none	GNG5
PTS	none	CEACAM6
PTS	none	PRSS3
PTS	none	RHEB
PTS	none	RPS27L
PTS	none	SMAD4
PTS	none	SPCS3
PTS	none	PSMB8
PTS	none	RPL18
PTS	none	C15orf48
PTS	none	SPATS2L
PTS	none	POLR2F
PTS	none	DPY30
PTS	none	B4GALT5
PTS	none	CD63
PTS	none	ARPC5
PTS	none	RAB11A
PTS	none	SHC1
PTS	none	HRCT1
PTS	none	MMADHC

PITPNA	none	PITPNA
CALM2	none	CALM2
CALM2	none	PPP2CB
CALM2	none	TNFRSF21
CALM2	none	BAIAP2L1
CALM2	none	TINAG
CALM2	none	CKS1B
CALM2	none	TCEB1
CALM2	none	NSL1
CALM2	none	CD164
CALM2	none	GNG5
CALM2	none	CEACAM6
CALM2	none	PRSS3
CALM2	none	RHEB
CALM2	none	RPS27L
CALM2	none	SPCS3
CALM2	none	PSMB8
CALM2	none	BUD31
CALM2	none	C15orf48
CALM2	none	SPATS2L
CALM2	none	POLR2F
CALM2	none	B4GALT5
CALM2	none	CD63
CALM2	none	ARPC5
CALM2	none	HRCT1
CALM2	none	MMADHC
PPP2CB	none	PPP2CB
PPP2CB	none	TINAG
PPP2CB	none	CKS1B
PPP2CB	none	NSL1
PPP2CB	none	GNG5
PPP2CB	none	JTB
PPP2CB	none	CMAS
PPP2CB	none	RPS9
PPP2CB	none	RHEB
PPP2CB	none	MUTYH
PPP2CB	none	RPS27L
PPP2CB	none	SMAD4
PPP2CB	none	MGST3
PPP2CB	none	PSMB8
PPP2CB	none	TARDBP
PPP2CB	none	RPL18
PPP2CB	none	NT5C3A
PPP2CB	none	SMIM20
PPP2CB	none	IL10RB
PPP2CB	none	ISX
PPP2CB	none	SPATS2L
PPP2CB	none	NDUFB8
PPP2CB	none	ANAPC16
PPP2CB	none	B4GALT5
PPP2CB	none	GNAS
PPP2CB	none	CBX3

PPP2CB	none	CD63
PPP2CB	none	ARPC5
PPP2CB	none	SMIM14
PPP2CB	none	ACTR10
PPP2CB	none	RAB11A
PPP2CB	none	SHC1
PPP2CB	none	HRCT1
PPP2CB	none	HIST1H2AC
PPP2CB	none	MMADHC
TIMM17B	none	TIMM17B
TIMM17B	none	HNRNPH3
TIMM17B	none	H3F3A
TIMM17B	none	COG1
POLR3GL	none	POLR3GL
POLR3GL	none	TNFRSF21
POLR3GL	none	VAPA
POLR3GL	none	MIER3
POLR3GL	none	SETD9
POLR3GL	none	CEACAM6
POLR3GL	none	ARL6IP5
POLR3GL	none	WAC-AS1
POLR3GL	none	ISX
POLR3GL	none	ANAPC16
POLR3GL	none	CD63
POLR3GL	none	DDX39B
POLR3GL	none	SMIM14
POLR3GL	none	ACTR10
POLR3GL	none	SHC1
TNFRSF2	none	TNFRSF21
TNFRSF2	none	BAIAP2L1
TNFRSF2	none	TINAG
TNFRSF2	none	CKS1B
TNFRSF2	none	VAPA
TNFRSF2	none	NSL1
TNFRSF2	none	CD164
TNFRSF2	none	MIER3
TNFRSF2	none	CEACAM6
TNFRSF2	none	RHEB
TNFRSF2	none	RPS27L
TNFRSF2	none	FGD6
TNFRSF2	none	WAC-AS1
TNFRSF2	none	DPY30
TNFRSF2	none	CD63
TNFRSF2	none	SHC1
TNFRSF2	none	MMADHC
TXNDC11	none	TXNDC11
TXNDC11	none	AGR3
TXNDC11	none	PPA1
TXNDC11	none	SRSF10
TXNDC11	none	SPINT2
TXNDC11	none	IFI27L1
BAIAP2L1	none	BAIAP2L1

BAIAP2L1	none	TINAG
BAIAP2L1	none	ZFAND5
BAIAP2L1	none	CD164
BAIAP2L1	none	RHEB
BAIAP2L1	none	SPCS3
BAIAP2L1	none	FGD6
BAIAP2L1	none	POLR2F
BAIAP2L1	none	ASNSD1
BAIAP2L1	none	WDR26
TINAG	none	TINAG
TINAG	none	CKS1B
TINAG	none	VAPA
TINAG	none	NSL1
TINAG	none	CD164
TINAG	none	GNG5
TINAG	none	CEACAM6
TINAG	none	PRSS3
TINAG	none	RHEB
TINAG	none	RPS27L
TINAG	none	SPCS3
TINAG	none	RPL18
TINAG	none	POLR2F
TINAG	none	DPY30
TINAG	none	B4GALT5
TINAG	none	CD63
TINAG	none	ARPC5
TINAG	none	VPS26B
TINAG	none	RAB11A
TINAG	none	SHC1
TINAG	none	HRCT1
TINAG	none	MMADHC
KDM4C	none	KDM4C
KDM4C	none	IKBKG
IKBKG	none	IKBKG
IKBKG	none	SYTL2
IKBKG	none	CTGF
IKBKG	none	COG1
LAPTM5	none	LAPTM5
LAPTM5	none	RGS2
CKS1B	none	CKS1B
CKS1B	none	VAPA
CKS1B	none	NSL1
CKS1B	none	CD164
CKS1B	none	GNG5
CKS1B	none	CEACAM6
CKS1B	none	CMAS
CKS1B	none	PRSS3
CKS1B	none	RHEB
CKS1B	none	RPS27L
CKS1B	none	SPCS3
CKS1B	none	PSMB8
CKS1B	none	RPL18

CKS1B	none	SPATS2L	
CKS1B	none	ANAPC16	
CKS1B	none	DPY30	
CKS1B	none	B4GALT5	
CKS1B	none	CD63	
CKS1B	none	ARPC5	
CKS1B	none	ACTR10	
CKS1B	none	RAB11A	
CKS1B	none	SHC1	
CKS1B	none	HRCT1	
CKS1B	none	MMADHC	
DOCK1	none	DOCK1	
DOCK1	none	CTSS	
09/01/14	none		09/01/14
09/01/14	none	U2AF1	
TSPO	none	TSPO	
TSPO	none	U2AF1	
TSPO	none	TCEB1	
TSPO	none	CD164	
TSPO	none	CLCA4	
TSPO	none	ITM2B	
TSPO	none	TICAM1	
TSPO	none	ACP1	
TSPO	none	GAPDH	
TSPO	none	GTF2B	
TSPO	none	NDUFB8	
TSPO	none	CD63	
TSPO	none	PMAIP1	
VAPA	none	VAPA	
VAPA	none	MIER3	
VAPA	none	LXN	
VAPA	none	SETD9	
VAPA	none	CEACAM6	
VAPA	none	RPS27L	
VAPA	none	RPL18	
VAPA	none	ARL6IP5	
VAPA	none	ISX	
VAPA	none	ANAPC16	
VAPA	none	HPGD	
VAPA	none	CD63	
VAPA	none	ARPC5	
VAPA	none	VPS26B	
VAPA	none	SMIM14	
VAPA	none	ACTR10	
VAPA	none	RAB11A	
VAPA	none	SHC1	
VAPA	none	HIST1H2AC	
CEBPB	none	CEBPB	
CEBPB	none	USP15	
CEBPB	none	KU-MEL-3	
CEBPB	none	CD55	
POLR2G	none	POLR2G	

POLR2G	none	INIP
HNRNPH3	none	HNRNPH3
HNRNPH3	none	SNRNP70
HNRNPH3	none	ITM2B
HNRNPH3	none	GTF2B
HNRNPH3	none	COG1
HNRNPH3	none	PMAIP1
PSMB3	none	PSMB3
PSMB3	none	TMEM44
PSMB3	none	CLCA4
PSMB3	none	CAPZA2
PSMB3	none	SPCS3
PSMB3	none	PSMB8
PSMB3	none	RPL18
PSMB3	none	NT5C3A
PSMB3	none	SMIM20
PSMB3	none	C15orf48
PSMB3	none	SPATS2L
PSMB3	none	POLR2F
PSMB3	none	CTSS
PSMB3	none	MCTP2
PSMB3	none	B4GALT5
PSMB3	none	CBX3
PSMB3	none	ARPC5
PSMB3	none	RAB11A
TMEM44	none	TMEM44
TMEM44	none	PSMB8
TMEM44	none	TMED9
TMEM44	none	NT5C3A
TMEM44	none	BUD31
TMEM44	none	ACP1
TMEM44	none	C15orf48
TMEM44	none	SPATS2L
TMEM44	none	MCTP2
TMEM44	none	B4GALT5
TMEM44	none	RBM22
TMEM44	none	CBX3
TMEM44	none	CD63
U2AF1	none	U2AF1
U2AF1	none	MGEA5
U2AF1	none	ANXA3
U2AF1	none	ACP1
U2AF1	none	CD63
U2AF1	none	PMAIP1
MGEA5	none	MGEA5
MGEA5	none	ANXA3
MGEA5	none	SMAP2
MGEA5	none	CAST
MGEA5	none	SERPINB1
MGEA5	none	CTSS
MGEA5	none	HSH2D
MGEA5	none	ZNF879

MGEA5	none	SEC14L1
TCEB1	none	TCEB1
TCEB1	none	OSER1
TCEB1	none	PRSS3
TCEB1	none	BUD31
TCEB1	none	GAPDH
TCEB1	none	C15orf48
ZFAND5	none	ZFAND5
ZFAND5	none	ARL5B
ZFAND5	none	POLR2F
ZFAND5	none	WDR26
HILPDA	none	HILPDA
HILPDA	none	CD55
UBE2DNL	none	UBE2DNL
UBE2DNL	none	BRIP1
C6orf211	none	C6orf211
C6orf211	none	KIAA1598
C6orf211	none	GOLM1
NSL1	none	NSL1
NSL1	none	GNG5
NSL1	none	CLCA4
NSL1	none	JTB
NSL1	none	ITM2B
NSL1	none	CMAS
NSL1	none	RPS9
NSL1	none	RHEB
NSL1	none	RPS27L
NSL1	none	MGST3
NSL1	none	PSMB8
NSL1	none	TARDBP
NSL1	none	RPL18
NSL1	none	NT5C3A
NSL1	none	SPATS2L
NSL1	none	NDUFB8
NSL1	none	ANAPC16
NSL1	none	B4GALT5
NSL1	none	CBX3
NSL1	none	CD63
NSL1	none	ARPC5
NSL1	none	CAMLG
NSL1	none	SMIM14
NSL1	none	ACTR10
NSL1	none	RAB11A
NSL1	none	SHC1
NSL1	none	HRCT1
NSL1	none	MMADHC
SLC26A3	none	SLC26A3
SLC26A3	none	ACTG1
SLC26A3	none	SDCBP2
RPS15	none	RPS15
RPS15	none	FOXJ3
AGR3	none	AGR3

AGR3	none	SRSF10
AGR3	none	SPINT2
AGR3	none	HPGD
ZFP36L1	none	ZFP36L1
ZFP36L1	none	KU-MEL-3
CD164	none	CD164
CD164	none	GNG5
CD164	none	CLCA4
CD164	none	SETD9
CD164	none	RHEB
CD164	none	SPCS3
CD164	none	PSMB8
CD164	none	RPL18
CD164	none	POLR2F
CD164	none	KRTCAP3
CD164	none	HPGD
CD164	none	B4GALT5
CD164	none	ARPC5
CD164	none	RAB11A
CD164	none	DHRS7
CD164	none	MMADHC
CD164	none	C3orf52
ANXA3	none	ANXA3
ANXA3	none	OSER1
ANXA3	none	CAST
ANXA3	none	SERPINB1
SERPINH:	none	SERPINH1
SERPINH:	none	GLS
MIER3	none	MIER3
MIER3	none	CMAS
MIER3	none	ARL6IP5
MIER3	none	WAC-AS1
MIER3	none	ISX
MIER3	none	ANAPC16
MIER3	none	ARRDC4
MIER3	none	CD63
MIER3	none	DDX39B
MIER3	none	SMIM14
MIER3	none	ACTR10
MIER3	none	SHC1
SNRNP70	none	SNRNP70
SNRNP70	none	GTF2B
SNRNP70	none	STK38L
SNRNP70	none	COG1
SNRNP70	none	TRAPPC10
SNRNP70	none	PMAIP1
LY86	none	LY86
LY86	none	STPG2
LY86	none	FOXP1
LY86	none	GLCCI1
GNG5	none	GNG5
GNG5	none	CLCA4

GNG5	none	JTB
GNG5	none	ITM2B
GNG5	none	CMAS
GNG5	none	PRSS3
GNG5	none	RPS9
GNG5	none	RHEB
GNG5	none	RPS27L
GNG5	none	MGST3
GNG5	none	PSMB8
GNG5	none	NT5C3A
GNG5	none	BUD31
GNG5	none	GAPDH
GNG5	none	SPATS2L
GNG5	none	NDUFB8
GNG5	none	B4GALT5
GNG5	none	RBM22
GNG5	none	CBX3
GNG5	none	CD63
GNG5	none	ARPC5
GNG5	none	ACTR10
GNG5	none	RAB11A
GNG5	none	SGK1
GNG5	none	SHC1
GNG5	none	HRCT1
GNG5	none	MMADHC
IFRD1	none	IFRD1
IFRD1	none	LAPTM4A
GOLGA4	none	GOLGA4
GOLGA4	none	SMAD4
GOLGA4	none	BTBD3
LXN	none	LXN
LXN	none	ZSWIM2
LXN	none	SETD9
LXN	none	ANAPC16
LXN	none	HPGD
LXN	none	VPS26B
LXN	none	SHC1
OSER1	none	OSER1
OSER1	none	SERPINB1
OSER1	none	AREG
CLCA4	none	CLCA4
CLCA4	none	CAPZA2
CLCA4	none	ITM2B
CLCA4	none	RHEB
CLCA4	none	SPCS3
CLCA4	none	PSMB8
CLCA4	none	NT5C3A
CLCA4	none	SMIM20
CLCA4	none	ACP1
CLCA4	none	SPATS2L
CLCA4	none	CTSS
CLCA4	none	B4GALT5

CLCA4	none	CBX3
CLCA4	none	CD63
CLCA4	none	ARPC5
CLCA4	none	CAMLG
CLCA4	none	SRSF9
CLCA4	none	RAB11A
CLCA4	none	MMADHC
JTB	none	JTB
JTB	none	LYPD8
JTB	none	CMAS
JTB	none	RPS9
JTB	none	CTGF
JTB	none	MGST3
JTB	none	TARDBP
JTB	none	IL10RB
JTB	none	ISX
JTB	none	DDX39B
JTB	none	COG1
JTB	none	CAMLG
JTB	none	SMIM14
JTB	none	ACTR10
JTB	none	HECA
JTB	none	HRCT1
ZSWIM2	none	ZSWIM2
ZSWIM2	none	CEACAM6
ZSWIM2	none	DPY30
ZSWIM2	none	VPS26B
ZSWIM2	none	ZFAND2A
SETD9	none	SETD9
SETD9	none	ARL6IP5
SETD9	none	HPGD
SETD9	none	VPS26B
SMAP2	none	SMAP2
SMAP2	none	CTSS
SMAP2	none	HSH2D
STPG2	none	STPG2
CAPZA2	none	CAPZA2
CAPZA2	none	SPCS3
CAPZA2	none	PSMB8
CAPZA2	none	NT5C3A
CAPZA2	none	SMIM20
CAPZA2	none	POLR2F
CAPZA2	none	PAK1
CAPZA2	none	CTSS
CAPZA2	none	B4GALT5
CAPZA2	none	ARPC5
CAPZA2	none	SRSF9
CAPZA2	none	ACTR10
CAPZA2	none	RAB11A
CAPZA2	none	MMADHC
ZCCHC24	none	ZCCHC24
ZCCHC24	none	HSFY1

ACTG1	none	ACTG1
ACTG1	none	RAB1A
ITM2B	none	ITM2B
ITM2B	none	RPS9
ITM2B	none	ACP1
ITM2B	none	GAPDH
ITM2B	none	SPATS2L
ITM2B	none	GTF2B
ITM2B	none	NDUFB8
ITM2B	none	RBM22
ITM2B	none	CBX3
ITM2B	none	CD63
ITM2B	none	COG1
ITM2B	none	SGK1
ITM2B	none	HRCT1
ITM2B	none	PMAIP1
CEACAM6	none	CEACAM6
CEACAM6	none	RHEB
CEACAM6	none	RPS27L
CEACAM6	none	WAC-AS1
CEACAM6	none	DPY30
CEACAM6	none	CD63
CEACAM6	none	VPS26B
CEACAM6	none	ZFAND2A
CEACAM6	none	SHC1
RBPJ	none	RBPJ
RBPJ	none	COG1
AKT3	none	AKT3
AKT3	none	OAZ2
AKT3	none	ACTR10
LYPD8	none	LYPD8
LYPD8	none	CMAS
LYPD8	none	ISX
LYPD8	none	PBLD
LYPD8	none	ACTR10
CMAS	none	CMAS
CMAS	none	RPS27L
CMAS	none	MGST3
CMAS	none	TARDBP
CMAS	none	ISX
CMAS	none	ANAPC16
CMAS	none	CD63
CMAS	none	DDX39B
CMAS	none	CAMLG
CMAS	none	SMIM14
CMAS	none	ACTR10
CMAS	none	SHC1
CMAS	none	HRCT1
CMAS	none	MMADHC
TICAM1	none	TICAM1
TICAM1	none	SRSF9
PRSS3	none	PRSS3

PRSS3	none	RHEB
PRSS3	none	SPCS3
PRSS3	none	BUD31
PRSS3	none	GAPDH
PRSS3	none	C15orf48
PRSS3	none	SPATS2L
PRSS3	none	B4GALT5
PRSS3	none	CD63
RPS9	none	RPS9
RPS9	none	CTGF
RPS9	none	MGST3
RPS9	none	TARDBP
RPS9	none	IL10RB
RPS9	none	GTF2B
RPS9	none	NDUFB8
RPS9	none	CBX3
RPS9	none	CD63
RPS9	none	COG1
RPS9	none	HRCT1
OAZ2	none	OAZ2
OAZ2	none	MUTYH
OAZ2	none	CTGF
OAZ2	none	NT5C3A
OAZ2	none	SMIM20
OAZ2	none	SFT2D1
OAZ2	none	CBX3
OAZ2	none	DDX39B
OAZ2	none	ARPC5
OAZ2	none	SMIM14
OAZ2	none	ACTR10
OAZ2	none	RAB11A
OAZ2	none	HECA
RHEB	none	RHEB
RHEB	none	RPS27L
RHEB	none	SPCS3
RHEB	none	PSMB8
RHEB	none	RPL18
RHEB	none	NT5C3A
RHEB	none	BUD31
RHEB	none	C15orf48
RHEB	none	SPATS2L
RHEB	none	POLR2F
RHEB	none	B4GALT5
RHEB	none	CD63
RHEB	none	ARPC5
RHEB	none	RAB11A
RHEB	none	SHC1
RHEB	none	HRCT1
RHEB	none	MMADHC
MUTYH	none	MUTYH
MUTYH	none	CTGF
MUTYH	none	MDK

MUTYH	none	MGST3
MUTYH	none	TARDBP
MUTYH	none	SFT2D1
MUTYH	none	IL10RB
MUTYH	none	ANAPC16
MUTYH	none	GNAS
MUTYH	none	CBX3
MUTYH	none	SMIM14
MUTYH	none	ACTR10
MUTYH	none	SHC1
MUTYH	none	HRCT1
MUTYH	none	HIST1H2AC
SYTL2	none	SYTL2
CTGF	none	CTGF
CTGF	none	MDK
CTGF	none	TARDBP
CTGF	none	IL10RB
CTGF	none	DDX39B
CTGF	none	COG1
CTGF	none	HECA
MDK	none	MDK
MDK	none	TARDBP
MDK	none	IL10RB
RPS27L	none	RPS27L
RPS27L	none	SMAD4
RPS27L	none	MGST3
RPS27L	none	PSMB8
RPS27L	none	RPL18
RPS27L	none	NT5C3A
RPS27L	none	SMIM20
RPS27L	none	SPATS2L
RPS27L	none	ANAPC16
RPS27L	none	B4GALT5
RPS27L	none	CBX3
RPS27L	none	CD63
RPS27L	none	ARPC5
RPS27L	none	SMIM14
RPS27L	none	ACTR10
RPS27L	none	RAB11A
RPS27L	none	SHC1
RPS27L	none	HRCT1
RPS27L	none	HIST1H2AC
RPS27L	none	MMADHC
SMAD4	none	SMAD4
SMAD4	none	MGST3
SMAD4	none	ANAPC16
SMAD4	none	GNAS
SMAD4	none	HRCT1
SMAD4	none	HIST1H2AC
MGST3	none	MGST3
MGST3	none	TARDBP
MGST3	none	IL10RB

MGST3	none	ANAPC16
MGST3	none	GNAS
MGST3	none	CBX3
MGST3	none	CD63
MGST3	none	SMIM14
MGST3	none	ACTR10
MGST3	none	SGK1
MGST3	none	HRCT1
MGST3	none	HIST1H2AC
CAST	none	CAST
CAST	none	SERPINB1
CAST	none	LAPTM4A
SPCS3	none	SPCS3
SPCS3	none	PSMB8
SPCS3	none	NT5C3A
SPCS3	none	BUD31
SPCS3	none	C15orf48
SPCS3	none	SPATS2L
SPCS3	none	POLR2F
SPCS3	none	PAK1
SPCS3	none	CTSS
SPCS3	none	B4GALT5
SPCS3	none	ARPC5
SPCS3	none	RAB11A
SPCS3	none	MMADHC
PSMB8	none	PSMB8
PSMB8	none	RPL18
PSMB8	none	NT5C3A
PSMB8	none	SMIM20
PSMB8	none	BUD31
PSMB8	none	ACP1
PSMB8	none	C15orf48
PSMB8	none	SPATS2L
PSMB8	none	POLR2F
PSMB8	none	CTSS
PSMB8	none	B4GALT5
PSMB8	none	RBM22
PSMB8	none	CBX3
PSMB8	none	CD63
PSMB8	none	ARPC5
PSMB8	none	ACTR10
PSMB8	none	RAB11A
PSMB8	none	HRCT1
PSMB8	none	MMADHC
TARDBP	none	TARDBP
TARDBP	none	IL10RB
TARDBP	none	ANAPC16
TARDBP	none	GNAS
TARDBP	none	CBX3
TARDBP	none	DDX39B
TARDBP	none	COG1
TARDBP	none	SMIM14

TARDBP	none	ACTR10
TARDBP	none	HRCT1
TARDBP	none	HIST1H2AC
RPL18	none	RPL18
RPL18	none	NT5C3A
RPL18	none	SMIM20
RPL18	none	SPATS2L
RPL18	none	ANAPC16
RPL18	none	HPGD
RPL18	none	B4GALT5
RPL18	none	CBX3
RPL18	none	CD63
RPL18	none	ARPC5
RPL18	none	SMIM14
RPL18	none	ACTR10
RPL18	none	RAB11A
RPL18	none	SHC1
RPL18	none	HRCT1
RPL18	none	HIST1H2AC
RPL18	none	MMADHC
SRSF10	none	SRSF10
SRSF10	none	SPINT2
SRSF10	none	IFI27L1
TMED9	none	TMED9
TMED9	none	C15orf48
TMED9	none	SPATS2L
TMED9	none	POLR2F
TMED9	none	ATP5F1
TMED9	none	RAB1A
NT5C3A	none	NT5C3A
NT5C3A	none	SMIM20
NT5C3A	none	SPATS2L
NT5C3A	none	CTSS
NT5C3A	none	B4GALT5
NT5C3A	none	CBX3
NT5C3A	none	ARPC5
NT5C3A	none	ACTR10
NT5C3A	none	RAB11A
NT5C3A	none	HRCT1
NT5C3A	none	HIST1H2AC
NT5C3A	none	MMADHC
FGD6	none	FGD6
FGD6	none	WAC-AS1
FGD6	none	ASNSD1
ARL6IP5	none	ARL6IP5
ARL6IP5	none	WAC-AS1
ARL6IP5	none	ISX
ARL6IP5	none	ANAPC16
ARL6IP5	none	ARRDC4
ARL6IP5	none	CD63
ARL6IP5	none	SMIM14
ARL6IP5	none	ACTR10

ARL6IP5	none	SHC1
WAC-AS1	none	WAC-AS1
WAC-AS1	none	DPY30
WAC-AS1	none	ARRDC4
WAC-AS1	none	CD63
WAC-AS1	none	VPS26B
SMIM20	none	SMIM20
SMIM20	none	SPATS2L
SMIM20	none	B4GALT5
SMIM20	none	CBX3
SMIM20	none	ARPC5
SMIM20	none	SMIM14
SMIM20	none	ACTR10
SMIM20	none	RAB11A
SMIM20	none	SHC1
SMIM20	none	HIST1H2AC
SMIM20	none	MMADHC
BUD31	none	BUD31
BUD31	none	GAPDH
BUD31	none	C15orf48
BUD31	none	SPATS2L
BUD31	none	POLR2F
BUD31	none	B4GALT5
BUD31	none	RBM22
BUD31	none	CD63
ACP1	none	ACP1
ACP1	none	SPATS2L
ACP1	none	GTF2B
ACP1	none	CTSS
ACP1	none	RBM22
ACP1	none	CBX3
ACP1	none	CD63
ACP1	none	SRSF9
ACP1	none	PMAIP1
SFT2D1	none	SFT2D1
SFT2D1	none	IL10RB
SFT2D1	none	TRAPPC10
IL10RB	none	IL10RB
IL10RB	none	ISX
IL10RB	none	ANAPC16
IL10RB	none	DDX39B
IL10RB	none	SMIM14
IL10RB	none	ACTR10
IL10RB	none	HIST1H2AC
SERPINB1	none	SERPINB1
SERPINB1	none	AREG
C15orf48	none	C15orf48
C15orf48	none	SPATS2L
C15orf48	none	POLR2F
C15orf48	none	B4GALT5
ISX	none	ISX
ISX	none	ANAPC16

ISX	none	CD63
ISX	none	DDX39B
ISX	none	SMIM14
ISX	none	ACTR10
ISX	none	SHC1
ISX	none	HIST1H2AC
ARL5B	none	ARL5B
ARL5B	none	WDR26
ARL5B	none	BNIP2
SPATS2L	none	SPATS2L
SPATS2L	none	B4GALT5
SPATS2L	none	RBM22
SPATS2L	none	CBX3
SPATS2L	none	CD63
SPATS2L	none	ARPC5
SPATS2L	none	RAB11A
SPATS2L	none	SGK1
SPATS2L	none	HRCT1
SPATS2L	none	MMADHC
MAPK9	none	MAPK9
MAPK9	none	WDR26
GTF2B	none	GTF2B
GTF2B	none	RBM22
GTF2B	none	CBX3
GTF2B	none	CD63
GTF2B	none	COG1
GTF2B	none	PMAIP1
POLR2F	none	POLR2F
POLR2F	none	B4GALT5
NDUFB8	none	NDUFB8
NDUFB8	none	CD63
NDUFB8	none	SGK1
NDUFB8	none	HRCT1
ANAPC16	none	ANAPC16
ANAPC16	none	CD63
ANAPC16	none	DDX39B
ANAPC16	none	ARPC5
ANAPC16	none	SMIM14
ANAPC16	none	ACTR10
ANAPC16	none	RAB11A
ANAPC16	none	SHC1
ANAPC16	none	HRCT1
ANAPC16	none	HIST1H2AC
PLEKHO1	none	PLEKHO1
KRTCAP3	none	KRTCAP3
H3F3A	none	H3F3A
H3F3A	none	COG1
H3F3A	none	HECA
PAK1	none	PAK1
CTSS	none	CTSS
CTSS	none	HSH2D
CTSS	none	CAMLG

CTSS	none	SRSF9
CTSS	none	ACTR10
CTSS	none	INIP
CTSS	none	SEC14L1
CTSS	none	MMADHC
KIAA1598	none	KIAA1598
USP15	none	USP15
CCNL1	none	CCNL1
SPINT2	none	SPINT2
SPINT2	none	HPGD
MCTP2	none	MCTP2
MCTP2	none	INIP
HPGD	none	HPGD
HPGD	none	VPS26B
HPGD	none	RAB11A
HPGD	none	SHC1
HPGD	none	DHRS7
DPY30	none	DPY30
DPY30	none	CD63
DPY30	none	ZFAND2A
DPY30	none	ACTR10
NUDCD3	none	NUDCD3
NUDCD3	none	ZNF879
NUDCD3	none	PYGL
PBLD	none	PBLD
ASNSD1	none	ASNSD1
ARRDC4	none	ARRDC4
ARRDC4	none	CD63
B4GALT5	none	B4GALT5
B4GALT5	none	RBM22
B4GALT5	none	CBX3
B4GALT5	none	CD63
B4GALT5	none	ARPC5
B4GALT5	none	RAB11A
B4GALT5	none	HRCT1
B4GALT5	none	MMADHC
UBXN2B	none	UBXN2B
UBXN2B	none	BIRC3
EEF1A1	none	EEF1A1
TRAPPC2	none	TRAPPC2
TRAPPC2	none	BTBD3
TRAPPC2	none	GLS
PAIP2	none	PAIP2
PAIP2	none	STK38L
PAIP2	none	LOC150381
PAIP2	none	LACTB
SDCBP2	none	SDCBP2
RBM22	none	RBM22
RBM22	none	CBX3
RBM22	none	CD63
RBM22	none	SGK1
RBM22	none	PMAIP1

KU-MEL-3	none	KU-MEL-3
GNAS	none	GNAS
GNAS	none	SGK1
GNAS	none	HRCT1
BTBD3	none	BTBD3
HSH2D	none	HSH2D
STK38L	none	STK38L
STK38L	none	TRAPPC10
ATP5F1	none	ATP5F1
ATP5F1	none	LACTB
FOXJ3	none	FOXJ3
CBX3	none	CBX3
CBX3	none	CD63
CBX3	none	ARPC5
CBX3	none	ACTR10
CBX3	none	RAB11A
CBX3	none	TRAPPC10
CBX3	none	SGK1
CBX3	none	HRCT1
CBX3	none	HIST1H2AC
CBX3	none	MMADHC
CD63	none	CD63
CD63	none	ARPC5
CD63	none	ACTR10
CD63	none	RAB11A
CD63	none	SGK1
CD63	none	SHC1
CD63	none	HRCT1
CD63	none	SEC14L1
CD63	none	PMAIP1
CD63	none	MMADHC
DDX39B	none	DDX39B
DDX39B	none	CAMLG
DDX39B	none	SMIM14
DDX39B	none	ACTR10
DDX39B	none	HECA
WDR26	none	WDR26
COG1	none	COG1
COG1	none	HECA
HSFY1	none	HSFY1
ARPC5	none	ARPC5
ARPC5	none	SMIM14
ARPC5	none	ACTR10
ARPC5	none	RAB11A
ARPC5	none	SHC1
ARPC5	none	HRCT1
ARPC5	none	HIST1H2AC
ARPC5	none	MMADHC
VPS26B	none	VPS26B
RAB9A	none	RAB9A
RAB9A	none	DHRS7
FOXP1	none	FOXP1

CAMLG	none	CAMLG
CAMLG	none	ACTR10
CAMLG	none	MMADHC
SRSF9	none	SRSF9
ZFAND2A	none	ZFAND2A
SMIM14	none	SMIM14
SMIM14	none	ACTR10
SMIM14	none	RAB11A
SMIM14	none	SHC1
SMIM14	none	HRCT1
SMIM14	none	HIST1H2AC
BRIP1	none	BRIP1
BRIP1	none	PGS1
BRIP1	none	SEH1L
BRIP1	none	RPRD2
PGS1	none	PGS1
ZNF879	none	ZNF879
CD55	none	CD55
ACTR10	none	ACTR10
ACTR10	none	RAB11A
ACTR10	none	SHC1
ACTR10	none	HRCT1
ACTR10	none	HIST1H2AC
ACTR10	none	MMADHC
GLS	none	GLS
SEH1L	none	SEH1L
RAB11A	none	RAB11A
RAB11A	none	SHC1
RAB11A	none	HRCT1
RAB11A	none	HIST1H2AC
RAB11A	none	MMADHC
TRAPPC1	none	TRAPPC10
TRAPPC1	none	LOC150381
SGK1	none	SGK1
SGK1	none	HRCT1
SGK1	none	PMAIP1
INIP	none	INIP
ABCB1	none	ABCB1
ABCB1	none	RAB1A
HECA	none	HECA
SHC1	none	SHC1
SHC1	none	HRCT1
SHC1	none	HIST1H2AC
SHC1	none	MMADHC
HRCT1	none	HRCT1
HRCT1	none	HIST1H2AC
HRCT1	none	MMADHC
AREG	none	AREG
HIST1H2A	none	HIST1H2AC
ACTN4	none	ACTN4
DHRS7	none	DHRS7
DHRS7	none	IFI27L1

SEC14L1	none	SEC14L1
LAPTM4A	none	LAPTM4A
RAB1A	none	RAB1A
GOSR1	none	GOSR1
IFI27L1	none	IFI27L1
PYGL	none	PYGL
RGS2	none	RGS2
LOC15038	none	LOC150381
LOC15038	none	LACTB
GOLM1	none	GOLM1
PMAIP1	none	PMAIP1
GLCCI1	none	GLCCI1
BIRC3	none	BIRC3
BAG3	none	BAG3
LACTB	none	LACTB
RPRD2	none	RPRD2
MMADHC	none	MMADHC
C3orf52	none	C3orf52
BNIP2	none	BNIP2

Source	Type	Target
PEPD	none	PEPD
PEPD	none	IKBKG
PEPD	none	ELMOD3
PEPD	none	RPL18
PDE4B	none	PDE4B
PDE4B	none	MUTYH
PDE4B	none	PCTP
GLUL	none	GLUL
GLUL	none	CHP1
GLUL	none	CALM2
GLUL	none	JTB
GLUL	none	Streptococcus_parasanguinis
GLUL	none	Eubacterium_limosum
SS18L2	none	SS18L2
SS18L2	none	EIF5
SS18L2	none	PCTP
LTA4H	none	LTA4H
LTA4H	none	SMYD4
LTA4H	none	MLLT4
LTA4H	none	TRAF3IP2
LTA4H	none	AGFG2
SRP14	none	SRP14
SRP14	none	LRP8
SRP14	none	FYN
JHDM1D	none	JHDM1D
JHDM1D	none	ORMDL1
JHDM1D	none	ARL5B
JHDM1D	none	HIPK3
SMYD4	none	SMYD4
SMYD4	none	MLLT4
SMYD4	none	TRAF3IP2
SMYD4	none	AGFG2
FABP2	none	FABP2
FABP2	none	PPP2CB
FABP2	none	CEBPB
FABP2	none	RHEB
FABP2	none	RBL1
GADD45A	none	GADD45A
GADD45A	none	CHCHD2
GADD45A	none	ADI1
GADD45A	none	TSPO
GADD45A	none	GTF2B
GADD45A	none	ARPC5
GADD45A	none	HECA
GADD45A	none	CPA6
RPL7	none	RPL7
RPL7	none	NDUFB4
RPL7	none	RPS15
RPL7	none	ST3GAL1
RPL7	none	MDK
RPL7	none	GNAS

RPL7	none	UBE2D3
CHP1	none	CHP1
CHP1	none	CALM2
CHCHD2	none	CHCHD2
CHCHD2	none	ADI1
CHCHD2	none	PTS
CHCHD2	none	TSPO
CHCHD2	none	GTF2B
CHCHD2	none	ARPC5
CHCHD2	none	HECA
CHCHD2	none	CPA6
EIF4E2	none	EIF4E2
EIF4E2	none	ACTG1
EIF4E2	none	MGST3
EIF4E2	none	B4GALT5
LAMC2	none	LAMC2
LAMC2	none	CEBPB
UQCRH	none	UQCRH
UQCRH	none	BAIAP2L1
UQCRH	none	ZFP36L1
UQCRH	none	TICAM1
UQCRH	none	SMIM20
UQCRH	none	MGST2
UQCRH	none	RAB11A
VPS36	none	VPS36
VPS36	none	CEBPB
VPS36	none	SPATS2L
FXYD3	none	FXYD3
FXYD3	none	ZFAND2A
LRP8	none	LRP8
LRP8	none	LINC00938
LRP8	none	C6orf211
EIF5	none	EIF5
NDUFB4	none	NDUFB4
NDUFB4	none	CCDC92
NDUFB4	none	SERPINH1
NDUFB4	none	UBE2D3
PPP1CA	none	PPP1CA
PPP1CA	none	KIAA1551
PPP1CA	none	MLLT4
PPP1CA	none	GAPDH
PGK2	none	PGK2
PGK2	none	SETD9
PPARG	none	PPARG
PPARG	none	GNG5
PPARG	none	SMIM20
ORMDL1	none	ORMDL1
ORMDL1	none	FYN
ORMDL1	none	LINC00938
ORMDL1	none	C6orf211
ORMDL1	none	ELMOD3
ORMDL1	none	TARDBP

ORMDL1	none	AGTPBP1
ORMDL1	none	CBX5
ORMDL1	none	PAIP2
ORMDL1	none	STK38L
ORMDL1	none	HRCT1
ORMDL1	none	SECTM1
CLIC4	none	CLIC4
CLIC4	none	CCDC92
CLIC4	none	PCTP
FYN	none	FYN
FYN	none	CTSS
CCDC92	none	CCDC92
CCDC92	none	SERPINH1
CCDC92	none	PCTP
CCDC92	none	UBE2D3
LINC00938	none	LINC00938
LINC00938	none	C6orf211
LINC00938	none	ELMOD3
LINC00938	none	FTX
LINC00938	none	FERMT3
LINC00938	none	STK38L
ADI1	none	ADI1
ADI1	none	PTS
ADI1	none	GTF2B
ADI1	none	WDR26
KIAA1551	none	KIAA1551
KIAA1551	none	DMTF1
KIAA1551	none	GAPDH
SEC61B	none	SEC61B
SEC61B	none	TINAG
TDP2	none	TDP2
TDP2	none	TNFRSF21
TDP2	none	ZFAND5
TDP2	none	HILPDA
TDP2	none	SPATS2L
TDP2	none	PBLD
TDP2	none	RYBP
TDP2	none	SEC14L1
ANAPC11	none	ANAPC11
ANAPC11	none	BAIAP2L1
ANAPC11	none	TCEB1
ANAPC11	none	CD63
ANAPC11	none	MGST2
GPSM3	none	GPSM3
GPSM3	none	LYPD8
PTS	none	PTS
PTS	none	WDR26
CALM2	none	CALM2
CALM2	none	ZFP36L1
CALM2	none	CD63
CALM2	none	MGST2
CALM2	none	RAB11A

PPP2CB	none	PPP2CB
PPP2CB	none	CEBPB
TIMM17B	none	TIMM17B
TIMM17B	none	TMEM44
TIMM17B	none	MDK
TIMM17B	none	UTP20
TIMM17B	none	ATP5F1
TIMM17B	none	UBE2D3
TNFRSF21	none	TNFRSF21
TNFRSF21	none	CEBPB
TNFRSF21	none	ZFAND5
TNFRSF21	none	HECA
BAIAP2L1	none	BAIAP2L1
BAIAP2L1	none	TCEB1
BAIAP2L1	none	TICAM1
BAIAP2L1	none	MGST2
TINAG	none	TINAG
TINAG	none	SRSF10
TINAG	none	ARRDC4
KDM4C	none	KDM4C
KDM4C	none	FTX
IKBKG	none	IKBKG
IKBKG	none	FTX
IKBKG	none	IFI27L1
CKS1B	none	CKS1B
CKS1B	none	VAPA
CKS1B	none	CLCA4
CKS1B	none	RGS2
09/01/14	none	09/01/14
09/01/14	none	VAPA
09/01/14	none	MIER3
09/01/14	none	NDUFB8
TSPO	none	TSPO
TSPO	none	SMIM20
TSPO	none	ARPC5
TSPO	none	SGK1
TSPO	none	HECA
TSPO	none	CPA6
VAPA	none	VAPA
VAPA	none	MIER3
VAPA	none	CD63
CEBPB	none	CEBPB
CEBPB	none	CAPZA2
CEBPB	none	AKT3
CEBPB	none	RPS9
CEBPB	none	RHEB
CEBPB	none	IL10RB
CEBPB	none	SPATS2L
CEBPB	none	PGS1
CEBPB	none	CD55
CEBPB	none	GLS
CEBPB	none	AREG

CEBPB	none	SEC14L1
HNRNPH3	none	HNRNPH3
HNRNPH3	none	ITM2B
HNRNPH3	none	RPL18
HNRNPH3	none	NT5C3A
TMEM44	none	TMEM44
TMEM44	none	MDK
TMEM44	none	RPS27L
TMEM44	none	UTP20
TMEM44	none	ATP5F1
MGEA5	none	MGEA5
MGEA5	none	ACP1
TCEB1	none	TCEB1
TCEB1	none	LYPD8
TCEB1	none	CD63
TCEB1	none	MGST2
MLLT4	none	MLLT4
MLLT4	none	GAPDH
MLLT4	none	TRAF3IP2
MLLT4	none	AGFG2
ZFAND5	none	ZFAND5
ZFAND5	none	HILPDA
ZFAND5	none	TICAM1
ZFAND5	none	B4GALT5
ZFAND5	none	SGK1
ZFAND5	none	CPA6
HILPDA	none	HILPDA
HILPDA	none	TICAM1
HILPDA	none	RPS9
HILPDA	none	B4GALT5
HILPDA	none	SGK1
HILPDA	none	SEC14L1
C6orf211	none	C6orf211
C6orf211	none	HIPK3
C6orf211	none	STK38L
SLC26A3	none	SLC26A3
SLC26A3	none	AGR3
SLC26A3	none	SETD9
SLC26A3	none	KRTCAP3
SLC26A3	none	WDR26
SLC26A3	none	LAPTM4A
EDEM3	none	EDEM3
EDEM3	none	TSPAN2
EDEM3	none	CBX5
RPS15	none	RPS15
RPS15	none	ST3GAL1
RPS15	none	MDK
AGR3	none	AGR3
AGR3	none	SETD9
AGR3	none	KRTCAP3
AGR3	none	MAGI1
FNDC3B	none	FNDC3B

FNDC3B	none	AKT3
FNDC3B	none	CHST11
ZFP36L1	none	ZFP36L1
ZFP36L1	none	TICAM1
ZFP36L1	none	SMIM20
ZFP36L1	none	MGST2
ZFP36L1	none	RAB11A
ZFP36L1	none	SGK1
ANXA3	none	ANXA3
ANXA3	none	IFRD1
ANXA3	none	ST3GAL1
ANXA3	none	GNAS
ANXA3	none	RBL1
ANXA3	none	ACTR10
SERPINH1	none	SERPINH1
PARD3	none	PARD3
PARD3	none	SECTM1
MIER3	none	MIER3
MIER3	none	MGST3
MIER3	none	NDUFB8
MIER3	none	B4GALT5
SMARCC1	none	SMARCC1
SMARCC1	none	CTGF
SMARCC1	none	PLEKHO1
SMARCC1	none	PBLD
SMARCC1	none	SDCBP2
SNRNP70	none	SNRNP70
SNRNP70	none	PRSS3
SNRNP70	none	PPA1
SNRNP70	none	CD55
CKS2	none	CKS2
CKS2	none	FTX
LY86	none	LY86
LY86	none	SETD9
GNG5	none	GNG5
GNG5	none	SMIM20
IFRD1	none	IFRD1
IFRD1	none	ST3GAL1
CLCA4	none	CLCA4
JTB	none	JTB
JTB	none	SMIM14
JTB	none	ZNF318
TSPAN2	none	TSPAN2
SETD9	none	SETD9
SETD9	none	MAGI1
SETD9	none	LAPTM4A
CAPZA2	none	CAPZA2
CAPZA2	none	PRSS3
CAPZA2	none	BUD31
CAPZA2	none	CD55
ACTG1	none	ACTG1
ACTG1	none	MGST3

ST3GAL1	none	ST3GAL1
ST3GAL1	none	MUTYH
ST3GAL1	none	GNAS
ITM2B	none	ITM2B
ITM2B	none	NT5C3A
ITM2B	none	MAU2
ITM2B	none	DECR1
RBPJ	none	RBPJ
RBPJ	none	PLEKHO1
RBPJ	none	CBX3
RBPJ	none	CAMLG
AKT3	none	AKT3
LYPD8	none	LYPD8
LYPD8	none	CD63
TICAM1	none	TICAM1
TICAM1	none	B4GALT5
TICAM1	none	SGK1
TICAM1	none	CPA6
PRSS3	none	PRSS3
PRSS3	none	BUD31
PRSS3	none	HPGD
PRSS3	none	CD55
RPS9	none	RPS9
RPS9	none	RHEB
RPS9	none	SGK1
RHEB	none	RHEB
MUTYH	none	MUTYH
MUTYH	none	PCTP
MUTYH	none	UBXN2B
MUTYH	none	GNAS
MUTYH	none	ACTR10
MUTYH	none	PAX6
SYTL2	none	SYTL2
SYTL2	none	DPM1
CTGF	none	CTGF
CTGF	none	CCNL1
CTGF	none	ZFAND2A
MDK	none	MDK
MDK	none	RPS27L
MDK	none	CLASP2
MDK	none	UTP20
MDK	none	ATP5F1
MDK	none	UBE2D3
RPS27L	none	RPS27L
RPS27L	none	CLASP2
RPS27L	none	UTP20
RPS27L	none	ATP5F1
MAML2	none	MAML2
MAML2	none	GTF2B
PPA1	none	PPA1
PPA1	none	Anaerostipes_caccae
ELMOD3	none	ELMOD3

ELMOD3	none	AGTPBP1
ELMOD3	none	FTX
ELMOD3	none	STK38L
ELMOD3	none	ABCB1
TAF6L	none	TAF6L
TAF6L	none	CCNL1
MGST3	none	MGST3
MGST3	none	B4GALT5
CAST	none	CAST
CAST	none	TMED9
CAST	none	RBL1
SPCS3	none	SPCS3
SPCS3	none	FERMT3
SPCS3	none	C15orf48
SPCS3	none	CTSS
SPCS3	none	NAMPT
SPCS3	none	IFIT1
PCTP	none	PCTP
PCTP	none	ACP1
PCTP	none	UBE2D3
TARDBP	none	TARDBP
TARDBP	none	FERMT3
TARDBP	none	DDX39B
RPL18	none	RPL18
AGTPBP1	none	AGTPBP1
AGTPBP1	none	STK38L
FTX	none	FTX
FTX	none	SECTM1
CLASP2	none	CLASP2
CLASP2	none	GNAS
CLASP2	none	ACTR10
SRSF10	none	SRSF10
SRSF10	none	ARRDC4
TMED9	none	TMED9
DMTF1	none	DMTF1
DMTF1	none	GAPDH
DMTF1	none	HSH2D
NT5C3A	none	NT5C3A
NT5C3A	none	DECR1
SMIM20	none	SMIM20
SMIM20	none	CPA6
BUD31	none	BUD31
ACP1	none	ACP1
DPM1	none	DPM1
DPM1	none	PAIP2
SFT2D1	none	SFT2D1
SFT2D1	none	CTSS
GAPDH	none	GAPDH
GAPDH	none	Akkermansia_muciniphila
GAPDH	none	Streptococcus_parasanguinis
IL10RB	none	IL10RB
IL10RB	none	SPATS2L

IL10RB	none	PBLD
FERMT3	none	FERMT3
FERMT3	none	CTSS
FERMT3	none	STK38L
FERMT3	none	DDX39B
FERMT3	none	NAMPT
FERMT3	none	IFIT1
C15orf48	none	C15orf48
C15orf48	none	CTSS
C15orf48	none	CD63
C15orf48	none	IFIT1
ARL5B	none	ARL5B
ARL5B	none	HIPK3
SPATS2L	none	SPATS2L
SPATS2L	none	SEC14L1
GTF2B	none	GTF2B
GTF2B	none	HECA
MAU2	none	MAU2
MAU2	none	HECA
NDUFB8	none	NDUFB8
PLEKHO1	none	PLEKHO1
PLEKHO1	none	H3F3A
PLEKHO1	none	PBLD
PLEKHO1	none	SDCBP2
PLEKHO1	none	CAMLG
HIPK3	none	HIPK3
CRIM1	none	CRIM1
CRIM1	none	TPK1
CRIM1	none	SECTM1
CBX5	none	CBX5
CBX5	none	USP15
CBX5	none	PAIP2
KRTCAP3	none	KRTCAP3
H3F3A	none	H3F3A
CTSS	none	CTSS
CTSS	none	IFIT1
USP15	none	USP15
USP15	none	PAIP2
CCNL1	none	CCNL1
SPINT2	none	SPINT2
SPINT2	none	PBLD
SPINT2	none	RYBP
UTP20	none	UTP20
UTP20	none	ATP5F1
MAGI1	none	MAGI1
MAGI1	none	WDR26
MAGI1	none	LAPTM4A
MCTP2	none	MCTP2
MCTP2	none	PAIP2
HPGD	none	HPGD
HPGD	none	ARRDC4
DPY30	none	DPY30

DPY30	none	RBM22
PBLD	none	PBLD
PBLD	none	SDCBP2
PBLD	none	SEC14L1
PBLD	none	TJP1
ARRDC4	none	ARRDC4
B4GALT5	none	B4GALT5
B4GALT5	none	SGK1
TPK1	none	TPK1
UBXN2B	none	UBXN2B
PAIP2	none	PAIP2
CHST11	none	CHST11
SDCBP2	none	SDCBP2
SDCBP2	none	TJP1
RYBP	none	RYBP
RBM22	none	RBM22
RBM22	none	DDX39B
GNAS	none	GNAS
GNAS	none	ACTR10
HSH2D	none	HSH2D
RBL1	none	RBL1
STK38L	none	STK38L
ATP5F1	none	ATP5F1
ATP5F1	none	UBE2D3
CBX3	none	CBX3
CD63	none	CD63
CD63	none	MGST2
UBE2D3	none	UBE2D3
DDX39B	none	DDX39B
WDR26	none	WDR26
ARPC5	none	ARPC5
ARPC5	none	SGK1
ARPC5	none	CPA6
MGST2	none	MGST2
MGST2	none	RAB11A
CAMLG	none	CAMLG
ZFAND2A	none	ZFAND2A
SMIM14	none	SMIM14
SMIM14	none	ZNF880
SMIM14	none	ZNF318
PGS1	none	PGS1
TRAF3IP2	none	TRAF3IP2
TRAF3IP2	none	AGFG2
CD55	none	CD55
ACTR10	none	ACTR10
GLS	none	GLS
RAB11A	none	RAB11A
SGK1	none	SGK1
SGK1	none	CPA6
PAX6	none	PAX6
ABCB1	none	ABCB1
HECA	none	HECA

HECA	none	CPA6
NAMPT	none	NAMPT
HRCT1	none	HRCT1
AREG	none	AREG
IFIT1	none	IFIT1
SEC14L1	none	SEC14L1
LAPTM4A	none	LAPTM4A
ZNF880	none	ZNF880
IFI27L1	none	IFI27L1
ZNF318	none	ZNF318
RGS2	none	RGS2
SECTM1	none	SECTM1
CPA6	none	CPA6
TJP1	none	TJP1
AGFG2	none	AGFG2
DECR1	none	DECR1
XDH	none	Veillonella_atypica
TRIM35	none	Bifidobacterium_dentium
TRIM35	none	Bifidobacterium_pseudocatenulatum
TNF	none	Akkermansia_muciniphila
TNF	none	Ruminococcus_gnavus
TNF	none	Bifidobacterium_dentium
TNF	none	Klebsiella_unclassified
TNF	none	Klebsiella_pneumoniae
TNF	none	Bifidobacterium_breve
TNF	none	Haemophilus_parainfluenzae
TNF	none	Streptococcus_thermophilus
TNF	none	Veillonella_unclassified
TNF	none	Veillonella_parvula
TNF	none	Lactobacillus_casei
TNF	none	Eubacterium_limosum
TNF	none	Bifidobacterium_unclassified
TNF	none	Bifidobacterium_longum
TNF	none	Bifidobacterium_bifidum
TNF	none	Bifidobacterium_adolescentis
TNF	none	Bacteroides_unclassified
TNF	none	Escherichia_unclassified
TNF	none	Escherichia_coli
TLR4	none	Klebsiella_unclassified
TLR4	none	Klebsiella_pneumoniae
TLR4	none	Bifidobacterium_breve
TLR4	none	Veillonella_unclassified
TLR4	none	Veillonella_parvula
TLR4	none	Lactobacillus_casei
TLR4	none	Eubacterium_limosum
TLR4	none	Bifidobacterium_unclassified
TLR4	none	Bifidobacterium_longum
TLR4	none	Bifidobacterium_adolescentis
TLR4	none	Bacteroides_unclassified
TLR4	none	Escherichia_unclassified
TLR4	none	Escherichia_coli

TLR2	none	Klebsiella_unclassified
TLR2	none	Klebsiella_pneumoniae
TLR2	none	Bifidobacterium_breve
TLR2	none	Veillonella_unclassified
TLR2	none	Veillonella_parvula
TLR2	none	Lactobacillus_casei
TLR2	none	Bifidobacterium_unclassified
TLR2	none	Bifidobacterium_longum
TLR2	none	Bifidobacterium_bifidum
TLR2	none	Bifidobacterium_adolescentis
TLR2	none	Bifidobacterium_pseudocatenulatum
TLR2	none	Enterococcus_faecalis
TLR2	none	Bacteroides_unclassified
TLR2	none	Escherichia_unclassified
TLR2	none	Escherichia_coli
TGFB1	none	Bifidobacterium_dentium
TGFB1	none	Haemophilus_parainfluenzae
TF	none	Akkermansia_muciniphila
TDO2	none	Eubacterium_limosum
TDO2	none	Bifidobacterium_pseudocatenulatum
TALDO1	none	Veillonella_parvula
TALDO1	none	Bifidobacterium_pseudocatenulatum
SUMF2	none	Haemophilus_parainfluenzae
SI	none	Streptococcus_thermophilus
SI	none	Bifidobacterium_unclassified
SI	none	Streptococcus_salivarius
SI	none	Bifidobacterium_longum
SI	none	Bifidobacterium_bifidum
SI	none	Bifidobacterium_adolescentis
SI	none	Bacteroides_unclassified
SI	none	Escherichia_unclassified
SI	none	Escherichia_coli
SERPINF2	none	Streptococcus.infantarius
SERPINF2	none	Anaerostipes_caccae
SERPINF2	none	Veillonella_dispar
SERPINF2	none	Klebsiella_unclassified
SERPINF2	none	Klebsiella_pneumoniae
SERPINF2	none	Enterobacter_cloacae
SERPINF2	none	Veillonella_atypica
SERPINF2	none	Haemophilus_parainfluenzae
SERPINF2	none	Eggerthella_lenta
SERPINF2	none	Veillonella_unclassified
SERPINF2	none	Veillonella_parvula
SERPINF2	none	Lactobacillus_casei
SERPINF2	none	Bifidobacterium_unclassified
SERPINF2	none	Streptococcus_salivarius
SERPINF2	none	Bifidobacterium_bifidum
SERPINF2	none	Bifidobacterium_adolescentis
SERPINF2	none	Enterococcus_faecalis
SERPINF2	none	Bacteroides_unclassified

SDS	none	Veillonella_dispar
RAD51	none	Streptococcus_parasanguinis
PHIP	none	Bifidobacterium_pseudocatenulatum
PGA5	none	Bifidobacterium_dentium
PGA5	none	Bifidobacterium_pseudocatenulatum
NFKB1	none	Ruminococcus_gnavus
NFKB1	none	Bifidobacterium_breve
NFKB1	none	Lactobacillus_casei
NFKB1	none	Bifidobacterium_unclassified
NFKB1	none	Streptococcus_salivarius
NFKB1	none	Bifidobacterium_longum
NFKB1	none	Bifidobacterium_adolescentis
NFKB1	none	Bacteroides_unclassified
NFKB1	none	Escherichia_unclassified
NFKB1	none	Escherichia_coli
MYBL1	none	Veillonella_atypica
MUC5B	none	Streptococcus_parasanguinis
MUC5AC	none	Akkermansia_muciniphila
MUC5AC	none	Bifidobacterium_dentium
MUC5AC	none	Bifidobacterium_breve
MUC5AC	none	Ruminococcus_torques
MUC5AC	none	Eggerthella_lenta
MUC5AC	none	Veillonella_unclassified
MUC5AC	none	Veillonella_parvula
MUC5AC	none	Bifidobacterium_unclassified
MUC5AC	none	Streptococcus_salivarius
MUC5AC	none	Bifidobacterium_longum
MUC5AC	none	Bifidobacterium_bifidum
MUC5AC	none	Bifidobacterium_adolescentis
MUC5AC	none	Bifidobacterium_pseudocatenulatum
MUC5AC	none	Bacteroides_unclassified
MUC2	none	Akkermansia_muciniphila
MUC2	none	Ruminococcus_gnavus
MUC2	none	Streptococcus_parasanguinis
MUC2	none	Ruminococcus_torques
MPO	none	Bifidobacterium_adolescentis
MGAM	none	Klebsiella_unclassified
MGAM	none	Klebsiella_pneumoniae
MGAM	none	Bifidobacterium_breve
MGAM	none	Streptococcus_thermophilus
MGAM	none	Veillonella_unclassified
MGAM	none	Veillonella_parvula
MGAM	none	Lactobacillus_casei
MGAM	none	Bifidobacterium_unclassified
MGAM	none	Streptococcus_salivarius
MGAM	none	Bifidobacterium_longum
MGAM	none	Bifidobacterium_bifidum
MGAM	none	Bifidobacterium_adolescentis
MGAM	none	Bacteroides_unclassified
MGAM	none	Escherichia_unclassified

MGAM	none	Escherichia_coli
LYZ	none	Veillonella_dispar
LYZ	none	Klebsiella_unclassified
LYZ	none	Klebsiella_pneumoniae
LYZ	none	Enterobacter_cloacae
LYZ	none	Streptococcus_thermophilus
LYZ	none	Veillonella_unclassified
LYZ	none	Veillonella_parvula
LYZ	none	Lactobacillus_casei
LYZ	none	Bifidobacterium_unclassified
LYZ	none	Streptococcus_salivarius
LYZ	none	Bifidobacterium_longum
LYZ	none	Bifidobacterium_bifidum
LYZ	none	Bifidobacterium_adolescentis
LYZ	none	Enterococcus_faecalis
LYZ	none	Bacteroides_unclassified
LYZ	none	Escherichia_unclassified
LYZ	none	Escherichia_coli
LTF	none	Bifidobacterium_dentium
LTF	none	Klebsiella_unclassified
LTF	none	Klebsiella_pneumoniae
LTF	none	Bifidobacterium_breve
LTF	none	Haemophilus_parainfluenzae
LTF	none	Streptococcus_thermophilus
LTF	none	Bifidobacterium_unclassified
LTF	none	Bifidobacterium_longum
LTF	none	Bifidobacterium_bifidum
LTF	none	Bifidobacterium_adolescentis
LTF	none	Bifidobacterium_pseudocatenulatum
LTF	none	Enterococcus_faecalis
LTF	none	Bacteroides_unclassified
LTF	none	Escherichia_unclassified
LTF	none	Escherichia_coli
LPAR1	none	Veillonella_dispar
INS	none	Roseburia_intestinalis
INS	none	Akkermansia_muciniphila
IL8	none	Streptococcus.infantarius
IL8	none	Klebsiella_unclassified
IL8	none	Klebsiella_pneumoniae
IL8	none	Bifidobacterium_breve
IL8	none	Haemophilus_parainfluenzae
IL8	none	Lactobacillus_casei
IL8	none	Bifidobacterium_unclassified
IL8	none	Streptococcus_salivarius
IL8	none	Bifidobacterium_longum
IL8	none	Bifidobacterium_bifidum
IL8	none	Bifidobacterium_adolescentis
IL8	none	Roseburia_inulinivorans
IL8	none	Bacteroides_unclassified
IL8	none	Escherichia_unclassified

IL8	none	Escherichia_coli
IL6	none	Roseburia_intestinalis
IL6	none	Akkermansia_muciniphila
IL6	none	Klebsiella_unclassified
IL6	none	Klebsiella_pneumoniae
IL6	none	Haemophilus_parainfluenzae
IL6	none	Veillonella_unclassified
IL6	none	Veillonella_parvula
IL6	none	Lactobacillus_casei
IL6	none	Eubacterium_limosum
IL6	none	Bifidobacterium_unclassified
IL6	none	Bifidobacterium_longum
IL6	none	Bifidobacterium_bifidum
IL6	none	Bifidobacterium_adolescentis
IL6	none	Bifidobacterium_pseudocatenulatum
IL6	none	Bacteroides_unclassified
IL6	none	Escherichia_unclassified
IL6	none	Escherichia_coli
IL5	none	Bifidobacterium_dentium
IL5	none	Bifidobacterium_adolescentis
IL5	none	Bifidobacterium_pseudocatenulatum
IL4	none	Bifidobacterium_dentium
IL4	none	Bifidobacterium_breve
IL4	none	Haemophilus_parainfluenzae
IL4	none	Lactobacillus_casei
IL4	none	Eubacterium_limosum
IL4	none	Bifidobacterium_unclassified
IL4	none	Bifidobacterium_longum
IL4	none	Bifidobacterium_bifidum
IL4	none	Bifidobacterium_adolescentis
IL23A	none	Veillonella_parvula
IL1B	none	Klebsiella_unclassified
IL1B	none	Klebsiella_pneumoniae
IL1B	none	Bifidobacterium_breve
IL1B	none	Haemophilus_parainfluenzae
IL1B	none	Veillonella_unclassified
IL1B	none	Veillonella_parvula
IL1B	none	Lactobacillus_casei
IL1B	none	Eubacterium_limosum
IL1B	none	Bifidobacterium_unclassified
IL1B	none	Bifidobacterium_longum
IL1B	none	Bifidobacterium_bifidum
IL1B	none	Bifidobacterium_adolescentis
IL1B	none	Bacteroides_unclassified
IL1B	none	Escherichia_unclassified
IL1B	none	Escherichia_coli
IL1A	none	Veillonella_parvula
IL17A	none	Akkermansia_muciniphila
IL17A	none	Klebsiella_unclassified
IL17A	none	Klebsiella_pneumoniae

IL17A	none	Bifidobacterium_breve
IL17A	none	Lactobacillus_casei
IL17A	none	Bifidobacterium_unclassified
IL17A	none	Bifidobacterium_bifidum
IL17A	none	Bacteroides_unclassified
IL17A	none	Escherichia_unclassified
IL17A	none	Escherichia_coli
IL12B	none	Bifidobacterium_pseudocatenulatum
IL10	none	Akkermansia_muciniphila
IL10	none	Ruminococcus_gnavus
IL10	none	Bifidobacterium_dentium
IL10	none	Klebsiella_unclassified
IL10	none	Klebsiella_pneumoniae
IL10	none	Bifidobacterium_breve
IL10	none	Haemophilus_parainfluenzae
IL10	none	Streptococcus_thermophilus
IL10	none	Veillonella_unclassified
IL10	none	Veillonella_parvula
IL10	none	Lactobacillus_casei
IL10	none	Eubacterium_limosum
IL10	none	Bifidobacterium_unclassified
IL10	none	Bifidobacterium_longum
IL10	none	Bifidobacterium_bifidum
IL10	none	Bifidobacterium_adolescentis
IL10	none	Bifidobacterium_pseudocatenulatum
IL10	none	Enterococcus_faecalis
IL10	none	Bacteroides_unclassified
IL10	none	Escherichia_unclassified
IL10	none	Escherichia_coli
IGHE	none	Bifidobacterium_pseudocatenulatum
IGHA1	none	Streptococcus_parasanguinis
IFNG	none	Akkermansia_muciniphila
IFNG	none	Bifidobacterium_dentium
IFNG	none	Klebsiella_unclassified
IFNG	none	Klebsiella_pneumoniae
IFNG	none	Bifidobacterium_breve
IFNG	none	Haemophilus_parainfluenzae
IFNG	none	Streptococcus_thermophilus
IFNG	none	Veillonella_unclassified
IFNG	none	Veillonella_parvula
IFNG	none	Lactobacillus_casei
IFNG	none	Eubacterium_limosum
IFNG	none	Bifidobacterium_unclassified
IFNG	none	Bifidobacterium_longum
IFNG	none	Bifidobacterium_bifidum
IFNG	none	Bifidobacterium_adolescentis
IFNG	none	Bifidobacterium_pseudocatenulatum
IFNG	none	Bacteroides_unclassified
IFNG	none	Escherichia_unclassified
IFNG	none	Escherichia_coli

HSPD1	none	Bifidobacterium_dentium
HSPD1	none	Streptococcus_parasanguinis
HSPD1	none	Klebsiella_unclassified
HSPD1	none	Klebsiella_pneumoniae
HSPD1	none	Enterobacter_cloacae
HSPD1	none	Bifidobacterium_breve
HSPD1	none	Streptococcus_thermophilus
HSPD1	none	Lactobacillus_casei
HSPD1	none	Bifidobacterium_unclassified
HSPD1	none	Streptococcus_salivarius
HSPD1	none	Bifidobacterium_longum
HSPD1	none	Bifidobacterium_adolescentis
HSPD1	none	Bifidobacterium_pseudocatenulatum
HSPD1	none	Enterococcus_faecalis
HSPD1	none	Bacteroides_unclassified
HSPA1B	none	Veillonella_dispar
HSPA1B	none	Veillonella_atypica
GUSB	none	Streptococcus.infantarius
GUSB	none	Ruminococcus_gnavus
GUSB	none	Bifidobacterium_dentium
GUSB	none	Enterobacter_cloacae
GUSB	none	Bifidobacterium_breve
GUSB	none	Veillonella_unclassified
GUSB	none	Lactobacillus_casei
GUSB	none	Bifidobacterium_unclassified
GUSB	none	Bifidobacterium_longum
GUSB	none	Bifidobacterium_bifidum
GUSB	none	Bifidobacterium_pseudocatenulatum
GUSB	none	Bacteroides_unclassified
GUSB	none	Escherichia_unclassified
GUSB	none	Escherichia_coli
GPT	none	Eggerthella_lenta
GPT	none	Bifidobacterium_pseudocatenulatum
GLB1	none	Streptococcus.infantarius
GLB1	none	Bifidobacterium_dentium
GLB1	none	Streptococcus_parasanguinis
GLB1	none	Klebsiella_unclassified
GLB1	none	Klebsiella_pneumoniae
GLB1	none	Enterobacter_cloacae
GLB1	none	Bifidobacterium_breve
GLB1	none	Haemophilus_parainfluenzae
GLB1	none	Streptococcus_thermophilus
GLB1	none	Veillonella_unclassified
GLB1	none	Veillonella_parvula
GLB1	none	Lactobacillus_casei
GLB1	none	Bifidobacterium_unclassified
GLB1	none	Streptococcus_salivarius
GLB1	none	Bifidobacterium_longum
GLB1	none	Bifidobacterium_bifidum
GLB1	none	Bifidobacterium_adolescentis

GLB1	none	Bifidobacterium_pseudocatenulatum
GLB1	none	Enterococcus_faecalis
GLB1	none	Bacteroides_unclassified
GLB1	none	Escherichia_unclassified
GLB1	none	Escherichia_coli
GLA	none	Streptococcus.infantarius
FN1	none	Streptococcus.infantarius
FN1	none	Veillonella_unclassified
FN1	none	Veillonella_parvula
FN1	none	Bifidobacterium_adolescentis
F12	none	Roseburia_intestinalis
ENGASE	none	Ruminococcus_torques
DLL1	none	Bifidobacterium_breve
DLL1	none	Veillonella_atypica
DLL1	none	Veillonella_unclassified
DLL1	none	Veillonella_parvula
DLL1	none	Lactobacillus_casei
DLL1	none	Streptococcus_salivarius
DLL1	none	Bifidobacterium_bifidum
DLL1	none	Bifidobacterium_adolescentis
DLL1	none	Enterococcus_faecalis
DEFB1	none	Bifidobacterium_dentium
CRP	none	Streptococcus_parasanguinis
CRP	none	Haemophilus_parainfluenzae
CRP	none	Eggerthella_lenta
CRP	none	Veillonella_unclassified
CFTR	none	Eubacterium_limosum
CD83	none	Bifidobacterium_pseudocatenulatum
CD248	none	Haemophilus_parainfluenzae
CD14	none	Veillonella_unclassified
CD14	none	Veillonella_parvula
CD14	none	Eubacterium_limosum
CD14	none	Bifidobacterium_adolescentis
CAT	none	Anaerostipes_caccae
CAT	none	Bifidobacterium_dentium
CAT	none	Ruminococcus_torques
CAT	none	Haemophilus_parainfluenzae
CAT	none	Eggerthella_lenta
CAT	none	Veillonella_unclassified
CAT	none	Veillonella_parvula
CAT	none	Lactobacillus_casei
CAT	none	Bifidobacterium_unclassified
CAT	none	Streptococcus_salivarius
CAT	none	Bifidobacterium_bifidum
CAT	none	Bifidobacterium_adolescentis
CAT	none	Bifidobacterium_pseudocatenulatum
CAT	none	Enterococcus_faecalis
CAT	none	Bacteroides_unclassified
CALCOCO2	none	Enterobacter_cloacae
CALCOCO2	none	Bifidobacterium_breve

CALCOCO2	none	Lactobacillus_casei
CALCOCO2	none	Bifidobacterium_unclassified
CALCOCO2	none	Bifidobacterium_longum
CALCOCO2	none	Bifidobacterium_bifidum
CALCOCO2	none	Bifidobacterium_adolescentis
CALCOCO2	none	Enterococcus_faecalis
CALCOCO2	none	Bacteroides_unclassified
CALCOCO2	none	Escherichia_unclassified
CALCOCO2	none	Escherichia_coli
C19orf25	none	Veillonella_dispar
AMY2A	none	Bifidobacterium_dentium
AMY2A	none	Klebsiella_unclassified
AMY2A	none	Klebsiella_pneumoniae
AMY2A	none	Veillonella_atypica
AMY2A	none	Streptococcus_thermophilus
AMY2A	none	Veillonella_unclassified
AMY2A	none	Lactobacillus_casei
AMY2A	none	Bifidobacterium_unclassified
AMY2A	none	Streptococcus_salivarius
AMY2A	none	Bifidobacterium_longum
AMY2A	none	Bifidobacterium_bifidum
AMY2A	none	Bifidobacterium_adolescentis
AMY2A	none	Enterococcus_faecalis
AMY2A	none	Roseburia_inulinivorans
ALKBH1	none	Ruminococcus_torques
ALB	none	Veillonella_atypica
ALB	none	Eggerthella_lenta
ALB	none	Veillonella_parvula
